# Quinolinate promotes macrophage-induced immune tolerance in glioblastoma through the NMDAR/PPARγ signaling axis

**DOI:** 10.1038/s41467-023-37170-z

**Published:** 2023-03-16

**Authors:** Pravin Kesarwani, Shiva Kant, Yi Zhao, Antony Prabhu, Katie L. Buelow, C. Ryan Miller, Prakash Chinnaiyan

**Affiliations:** 1 Department of Radiation Oncology, Corewell Health East, Royal Oak, MI USA; 2 Department of Pathology, Division of Neuropathology, Heersink School of Medicine, University of Alabama at Birmingham, Birmingham, AL USA; 3 Oakland University William Beaumont School of Medicine, Royal Oak, MI USA

**Keywords:** Cancer metabolism, Tumour immunology, CNS cancer

## Abstract

There has been considerable scientific effort dedicated to understanding the biologic consequence and therapeutic implications of aberrant tryptophan metabolism in brain tumors and neurodegenerative diseases. A majority of this work has focused on the upstream metabolism of tryptophan; however, this has resulted in limited clinical application. Using global metabolomic profiling of patient-derived brain tumors, we identify the downstream metabolism of tryptophan and accumulation of quinolinate (QA) as a metabolic node in glioblastoma and demonstrate its critical role in promoting immune tolerance. QA acts as a metabolic checkpoint in glioblastoma by inducing NMDA receptor activation and Foxo1/PPARγ signaling in macrophages, resulting in a tumor supportive phenotype. Using a genetically-engineered mouse model designed to inhibit production of QA, we identify kynureninase as a promising therapeutic target to revert the potent immune suppressive microenvironment in glioblastoma. These findings offer an opportunity to revisit the biologic consequence of this pathway as it relates to oncogenesis and neurodegenerative disease and a framework for developing immune modulatory agents to further clinical gains in these otherwise incurable diseases.

## Abstract (web summary)

The upstream metabolism of tryptophan has been described as a metabolic node in glioblastoma. Here the authors show that the downstream metabolism of tryptophan, resulting in the accumulation of quinolinate in glioblastoma, contributes to pro-tumorigenic immune suppressive activation of macrophages.

## Introduction

The capacity of cancer cells to evolve mechanisms to evade the host immune system represents a requisite event for these cells to survive and continue to proliferate and metastasize^1^. Accordingly, investigations have identified numerous ways by which these cells actively sculpt the tumor microenvironment to promote immune tolerance. In brain tumors, tumor-associated macrophages (TAMs) have emerged as critical cells contributing towards their potent immune suppressive microenvironment^2^. Macrophages and microglial cells (resident macrophages) constitute over 30% of the immune fraction in glioblastoma (GBM) and, therefore, likely act as a major component of neuroinflammation and immune dysregulation in this malignancy^3,4^. Although refined in recent years, early research has classified TAMs as proinflammatory macrophages (M1 macrophages), responsible for anti-tumorigenic function and anti-inflammatory macrophages (alternatively activated M2 macrophages) contributing towards a pro-tumorigenic phenotype. Alternatively activated M2 macrophages are a type of TAM contributing towards immunosuppression, angiogenesis, and tumor progression and, therefore, represent an important target for immunotherapy^5–7^.

**Fig. 1:**
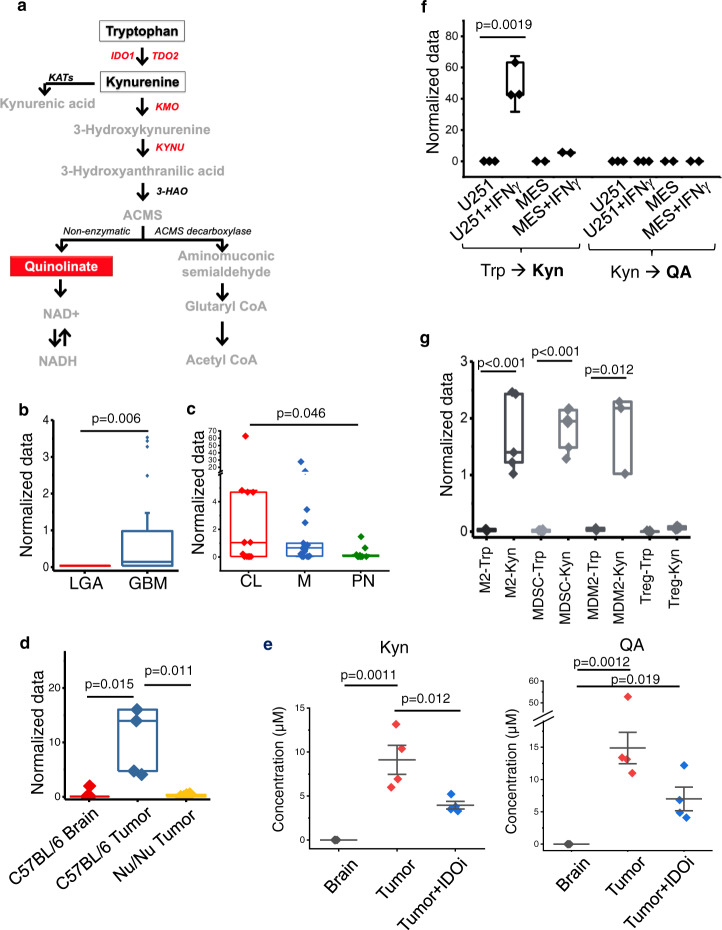
Quinolinate (QA) accumulation in glioblastoma (GBM). **a** Schematic of tryptophan (Trp) metabolism. **b** Metabolomic profiling performed on patient-derived low-grade astrocytoma (LGA; *n* = 28; red) and GBM (*n* = 80; blue) demonstrates differential accumulation of QA in GBM. All samples were biologically independent. **c** The relative accumulation of QA was evaluated in molecular subtypes of GBM (*n* = 46), classified as classical (CL, *n* = 13; red), mesenchymal (M, *n* = 21; blue), or proneural (PN, *n* = 12; green). Y axis was abridged at 4. **d** The murine GBM line TRP was grown orthotopically in C57BL/6 mice (*n* = 5; blue) and Nu/Nu mice (*n* = 6; yellow) and analyzed for QA and compared to normal murine brain (*n* = 5; red). All samples were biologically independent. **e** QA and kynurenine (Kyn) were quantified in normal brain (gray) and TRP tumors grown orthotopically in C57BL/6 mice with (blue) and without (red) the IDO inhibitor (IDOi) GDC-0919 (*n* = 3 biologically independent samples/group). **f** The upstream and downstream metabolism of tryptophan (Trp→Kyn and Kyn→QA, respectively) was evaluated in U251 (*n* = 3) and MES83 (*n* = 2) cells +/− IFNγ. All samples were biologically independent. **g** M2 macrophages (*n* = 6), myeloid-derived-suppressor cells (MDSC; *n* = 6), microglial-derived M2 cells (MDM2; *n* = 3), and regulatory T cells (Treg; *n* = 4) were isolated from C57BL/6 mice, cultured with Trp or Kyn, and analyzed for QA. All samples were biologically independent. Box plots represent interquartile range, line between data points represents mean, and whiskers represent SE (**b**, **c**, **d**, **f** and **g**). Line between data points represents mean, and whiskers represent SE (**e**). Statistics: Two-tailed Student’s *t*-test (**b**, **f**, and **g**), one-way ANOVA followed by Tukey’s multiple comparisons test (**c**, **d** and **e**). All tests were performed at 95% confidence interval. Source data are provided as a source data file.

Recent investigations have uncovered elegant strategies cancer cells use to promote a state of immune tolerance in the tumor microenvironment by exploiting conserved, metabolic signaling^8–11^. Aberrant tryptophan metabolism, and its metabolic intermediate kynurenine (Kyn), represents one such pathway that has received considerable attention (Fig. 1a). Tryptophan is metabolized to Kyn by the rate limiting enzymes indoleamine 2,3-dioxygenase 1 (IDO1) and tryptophan 2,3-dioxygenase (TDO)^12,13^ and contributes to an immune tolerant environment at many levels. Intriguingly, the most notable physiologic role of this metabolic pathway has been attributed to peripheral immune tolerance and fetal protection from maternal immune rejection in the placenta^14^. A variety of tumors, including GBM, have evolved mechanisms to co-opt this potent mechanism of suppression to evade the host immune system, which has been an active area of research with a particular focus on the immune-modulatory metabolite Kyn^12,13,15^.

Although accumulation of quinolinate (QA) has been both recognized and a focus of neuropathology for nearly four decades, its potential role in shaping the immune microenvironment has not been studied in detail^16–18^. As QA does not cross the blood brain barrier, concentrations are typically low (<100 nM) in the brain; however, its accumulation has been observed in a variety of pathologic states, including Alzheimer’s and Huntington’s disease, acquired immunodeficiency syndrome dementia complex, trauma, and meningitis^19–22^. Early studies in neuropathology demonstrated the accumulation of QA leads to neuronal death/dysfunction through several mechanisms, including N-methyl-D-aspartate (NMDA) receptor-mediated excitotoxicity, lipid peroxidation, and synthesis of nitric oxide^23–25^. However, its capacity to modulate other biological functions remains largely undefined. Recently, our group has identified the accumulation of QA in GBM, suggesting a contributory role in tumor growth.

In this work, we discover the accumulation of QA to be an important metabolic checkpoint contributing towards the immune suppressive microenvironment in GBM. We uncover a mechanism by which QA utilizes the NMDA-receptor to induce Foxo1/PPARγ transcription factor signaling in macrophages, resulting in a highly immune-suppressive M2-like state. Targeting this pathway demonstrates immune activation and anti-tumor activity, offering strong promise in GBM and a framework that may be applied for the treatment of a variety of neurodegenerative diseases where QA has been demonstrated to accumulate^19,26,27^.

## Results

### Quinolinate accumulation in glioblastoma

In our previous work, we performed cross-platform analyses coupling global metabolomic profiling with genomics in over 100 patient-derived brain tumors to provide a window into specific metabolic programs driving the aggressive phenotype of this malignancy in the context of its molecular architecture^9,28^. Through these studies, we identified the accumulation of QA, the downstream metabolic intermediate of tryptophan, in GBM, with a ~64-fold increase when compared to low-grade astrocytoma (LGA; Fig. 1b). We went on to determine if QA accumulation was a direct consequence of established molecular subtypes in GBM. Although outliers with particularly high levels of QA were only observed in isocitrate dehydrogenase 1 (IDH1) wild-type (WT) and O-6-methylguanine-DNA methyltransferase (MGMT) unmethylated tumors, two of the strongest prognostic factors in GBM^29,30^, collectively, levels of this metabolite were not significantly different between these subtypes (Suppl. Fig. 1). Next, using gene expression profiles generated from these matched samples, tumors were classified according to their molecular subtype: mesenchymal, classical, or proneural^31,32^. Interestingly, the accumulation of QA was specific to the mesenchymal and classical subtypes, when compared to proneural GBM (Fig. 1c). The accumulation of QA in GBM was further validated in an independent data set using immunohistochemical (IHC) staining (Suppl. Fig. 2).

We next sought to determine if QA accumulation was recapitulated in GBM preclinical models. QA was present when evaluated in cell lines derived from a genetically engineered mouse model (GEMM; TRP cell line^33,34^) grown intracranially in C57BL/6 J mice, but not in normal brain (Fig. 1d), which we went on to quantify (mean concentration ~20 µM; Fig. 1e). Interestingly, QA was not detected when evaluated in TRP tumors grown in immune deficient mice (Fig. 1d), suggesting immune cells may play a role in the intermediary metabolism of tryptophan and the accumulation of QA. As discussed above, targeting the upstream metabolism of tryptophan has been an active are of investigation. We therefore sought to determine how targeting the upstream metabolism of tryptophan may influence the accumulation of the downstream metabolic intermediate QA. Using the IDO1 inhibitor GDC-0919, we demonstrate that although a reduction is Kyn is observed, it is not complete (Fig. 1e), which may be attributed to parallel pathways a tumor may utilize to generate this metabolite independent of IDO1 or incomplete enzyme inhibition by this compound. Importantly, in a similar fashion, when evaluating for QA, although IDO1 inhibition resulted in a decrease in QA, biologically relevant concentrations were maintained in the tumor, suggesting a more specific, downstream enzyme may be a more relevant target to attenuate the accumulation of QA.

Although we and others have previously demonstrated that upon IFN-γ mediated activation, GBM cell lines are able to generate Kyn when cultured with tryptophan^10,13^, here we show that these tumor cells do not have the capacity to independently complete this metabolic pathway in its entirety, as QA was not present when GBM cell lines were cultured with either tryptophan or Kyn (Fig. 1f). As our above investigations suggested immune cells may play a role in the accumulation of QA, we evaluated the potential of immune cells typical to the GBM microenvironment, including M2 macrophages, myeloid derived suppressive cells (MDSCs), microglia derived M2-like macrophages (MDM2s), and regulatory T cells (Tregs), to generate QA. Interestingly, paradoxical to tumors, although myeloid derived immune cells and microglia did not have the capacity to metabolize tryptophan, they were able to metabolize Kyn to generate QA (Fig. 1g). We went on to validate these static findings using a flux-based approach (Suppl. Fig. 3). In GBM cells cultured with 15N2 labeled Trp, labeled Trp and Kyn were detected in both cells and media (termed conditioned media), yet no labeled QA was present. M2 macrophages were not able to generate Kyn or QA when cultured with 15N2 labeled Trp. However, high levels of labeled QA was detected in the media when M2 macrophages were cultured in GBM conditioned media, further supporting that QA is produced by a dynamic interaction between tumor cells and macrophages in a paracrine manner.

### QA contributes towards immune suppression by priming macrophage polarization towards an M2-like phenotype

Next, we sought to determine the biologic consequence of QA in GBM. As the role of QA’s upstream mediator Kyn in immune response has been well-described^10,12–14^, we evaluated for possible immune consequences of QA. As an initial investigation, we examined the potential for QA, along with its upstream and downstream metabolic intermediates, Kyn and NAD + respectively (Fig. 1a), to polarize and/or promote proliferation/differentiation of immune suppressive cells. Of the immune cells tested, Tregs, MDSCs, and macrophages, only macrophages appeared to be influenced by QA, resulting in a dramatic increase in differentiation from 47.4 ± 5.3% to 70.9 ± 7.8% (Suppl. Fig. 4a). This was not observed with Kyn or NAD + , suggesting a direct action of this metabolite rather than the consequence of its downstream metabolism. As an initial investigation, we extended findings to determine if QA contributed towards macrophage polarization by evaluating established markers differentiating M1 (CD80^hi^) and M2 (CD206 + ) macrophages. Although no changes in CD80^hi^ macrophages was observed with QA when polarizing macrophages towards the M1 phenotype (Suppl. Fig. 4b), a robust ~50% increase in the M2 macrophage marker CD206 + (38.2% to 56.7%) was observed (Fig. 3a), although CD209 + expression remained unchanged (Suppl. Fig. 4c). We went on to extend these findings, demonstrating QA significantly increases the immune-suppressive markers IL4Rα + and Arginase1 in macrophages (Fig. 3a), further supporting the concept that QA skews macrophage polarization towards an M2-like phenotype.

**Fig. 2:**
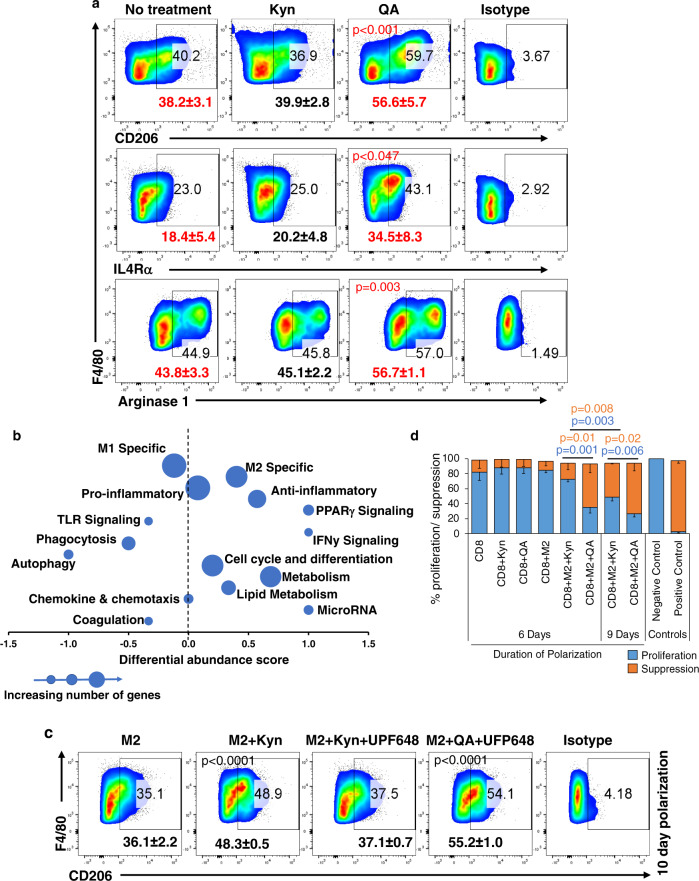
QA contributes towards immune suppression by priming monocyte polarization towards an M2-like phenotype. **a** Macrophages polarized towards the M2 phenotype ±Kyn or QA (20 µM) were gated for CD45 + F4/80 + CD11b + and analyzed for CD206 (*n* = 3 or 5/group as indicated in Source Data), IL-4Rα (*n* = 3/group), or Arginase 1 (*n* = 3/group) using flow-cytometry. Numbers represent mean ± SD. All samples were biologically independent. **b** M2 macrophages cultured in presence or absence of QA (20 µM) were flow-sorted for M2 macrophages (CD45 + CD11b + F4/80 + CD206 + ), and mRNA analyzed using Affymetrix arrays (Clariom™ D Assay, mouse; *n* = 5 biologically independent samples/group). Transcripts were classified into major pathways and a differential abundance (DA) score was calculated using mRNA transcripts significantly increased or decreased (Mann-Whitney U tests and Benjamini-Hochberg corrected *p*-value < 0.05) in the M2 + QA group compared to the M2 macrophage group. A score of 1 or −1 indicates all genes in a given pathway were increased or decreased, respectively. **c** Macrophages polarized towards the M2 phenotype ±Kyn or QA (20 µM) for an additional 3 day (10 days total, to allow for further metabolism/generation of QA from Kyn) ± the kynurenine 3-monooxygenase (KMO) inhibitor UFP648. Cells were gated for CD45 + F4/80 + CD11b + CD206 + using flow-cytometry. Numbers represent mean ± SD (*n* = 3 biologically independent samples/group). **d** To analyze the functional suppression of M2 macrophages, M2 cells were polarized ±Kyn or QA (20 µM) for 6 or 9 day (*n* = 3 biologically independent samples/group). Splenocytes from C57BL/6 mice were used for isolating CD8 + T cells using magnetic bead sorting. CFSE labeled CD8 + T cells were activated using plate-bound anti-CD3/CD28 antibody for 3 day in the presence or absence of M2 cells ±Kyn or QA. Data is represented as a bar graph (mean ± SD) demonstrating CFSE dilution (proliferation; blue) or suppression (orange). CFSE labeled CD8 + T cells without stimulation (anti-CD3/CD28 antibody) were used as a positive control of suppression. Unlabeled CD8 + T cells were used as a negative control for proliferation. Statistics: Two-tailed Student’s *t*-test (**a** and **d**), one-way ANOVA followed by Tukey’s multiple comparisons test (**c**). All tests were performed at 95% confidence interval. Source data are provided as a source data file.

As recent investigations have demonstrated the M1/M2 dichotomy of defining macrophages do not accurately recapitulate the complex and dynamic states of these cells, we extended investigations using gene expression profiling to gain a more comprehensive understanding of the observed QA-induced changes in the diverse action states and phenotypes of macrophages. A total of 221 genes were differentially regulated by QA in macrophages, which were classified into major pathways, and a differential abundance score (DA score) analysis was performed (Fig. 2b). A score of 1 and −1 indicates all genes in a given pathway were increased or decreased, respectively. Consistent with our initial findings, QA appeared to upregulate M2 macrophage-specific genes (Pparg, Klf4, Ptgs1, Gpr132^35–38^) when compared to M1-specific genes (Nfkb1, Traf1, Icam1)^39,40^. In addition, QA led to an upregulation of several anti-inflammatory genes (Pparg, Pilra, Gpr123^41–43^) and downregulation of autophagy genes (including Tlr7 and Igf3^44,45^) and phagocytosis markers (CD93) in macrophages. Collectively, these studies support the concept that QA confers an M2-like phenotype to macrophages.

As we have already established that macrophages have the unique ability to metabolize Kyn to QA, we hypothesized that rather than the standard 7 days schedule of polarization, a longer schedule (9–10 days) would provide these cells time to generate QA, which in turn, could influence macrophage plasticity. As predicted, although not as potent as QA, a longer schedule with Kyn did result in an increase in CD206 + macrophages. Importantly, these changes were mitigated by inhibiting the upstream biosynthetic enzyme kynurenine-3-monooxygenase (KMO; UPF648), in Kyn, but not in QA treated cells (Fig. 2c), further supporting the direct role QA plays in macrophage polarization. The potential for QA to modulate macrophage polarization was validated in the established murine macrophage cell line IC-21, which again resulted in a near doubling of CD206 + macrophages (Suppl. Fig. 5).

We went on to determine if the observed changes in macrophage markers translated to the capacity of these cells to functionally suppress CD8 + T-cell proliferation using CD8/M2 macrophage co-culture experiments. At the indicated CD8:M2 ratio (5:1), M2 macrophages and QA alone did not inhibit CD8 cell proliferation (Fig. 2d, Suppl. Fig. 6a). Consistent with polarization studies, M2 macrophages cultured in the presence of QA led to potent inhibition of CD8 + proliferation (35.1 ± 7.6%), while Kyn resulted in minimal changes (72.4 ± 2.9%) when tested in standard 6 days polarization conditions. When polarization was extended to 9 days (allowing time for M2 macrophages to metabolize Kyn to QA), inhibition of CD8 + proliferation was observed (48.9 ± 5.6%), albeit less than when cultured with QA (26.5 ± 4.5%). In addition to CD8 + proliferation, QA significantly diminished expression of granzyme B + in CD8 cells (Suppl. Fig. 6b) and its influence on polarization and proliferation was further validated when extended to MDM2 cells (Suppl. Fig. 7a/b).

### QA confers an M2-like phenotype to M1 macrophages and microglia

**Fig. 3:**
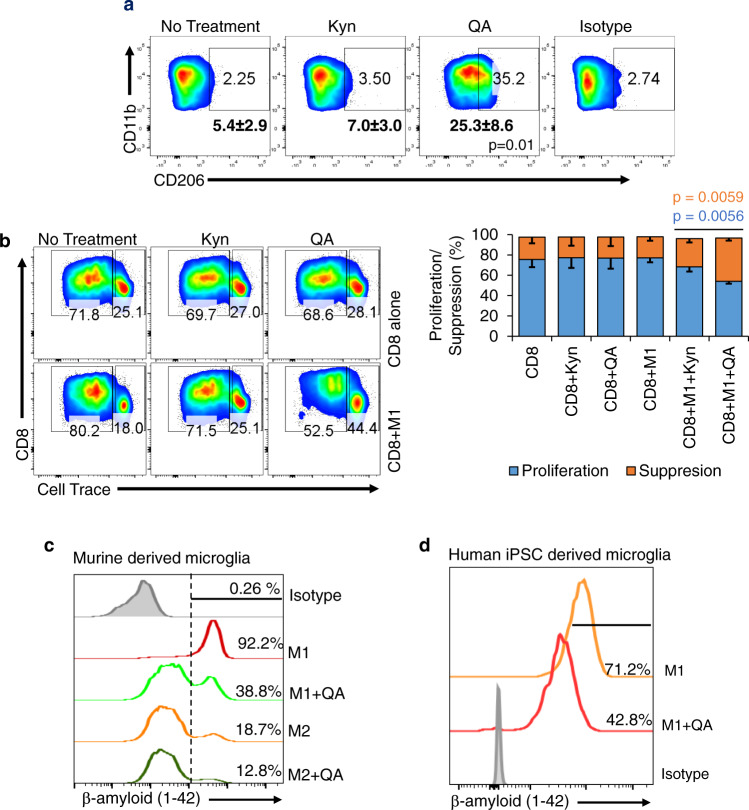
QA induces an immune suppressive phenotype to M1 macrophages and microglia. **a** M0 macrophages harvested from the bone marrow of C57BL/6 mice were polarized to M1 macrophages with LPS (100 ng/ml) and IFNγ (50 ng/ml) for 24–36 h ±Kyn or QA (20 µM) and gated for M2 macrophage markers (CD45 + F4/80 + CD11b + CD206 + ). Numbers represent mean ± SD (*n* = 3 biologically independent samples/group). **b** The functional suppression of M1 macrophages polarized + /- Kyn or QA was performed using CD8/macrophage co-culture experiments with CFSE dilution, as described in Fig. 2. Bar graph demonstrate mean and SD (*n* = 3 biologically independent samples/group). **c** Murine-derived microglia were matured in the presence of GM-CSF conditioning media and polarized towards the M1 phenotype (LPS + IFNγ) with (green) or without (red) QA or the M2 phenotype (IL4 + IL13) with (dark green) or without (orange) QA. Cells were pulsed with green florescent β-amyloid (1-42) peptide and analyzed for phagocytosis of this peptide at 16 h by flow cytometry. **d** Similar studies were performed using human induced pluripotent stem cell (iPSC) derived microglia and polarized towards the M1 phenotype (LPS + IFNγ) with (red) or without (orange) QA. Data is representative of three biologically independent experiments (**c**, **d**). Statistics: One-way ANOVA followed by Tukey’s multiple comparisons test (**a**, **b**), two-tailed Student’s *t*-test (**b**). All tests were performed at 95% confidence interval. Source data are provided as a source data file.

As we demonstrated QA plays a role in polarizing macrophages towards an M2-like phenotype with immune suppressive properties, we extended investigations to determine if this metabolite influenced M1 macrophage polarization and/or phenotype. Although as discussed above, QA did not appear to influence M1 polarization (CD45 + CD11b + F4/80 + CD80hi, Suppl. Fig. 4b), intriguingly, it did confer an M2-like molecular phenotype to these cells, with the percentage of M1 macrophages expressing the M2 macrophage marker CD206 + dramatically increasing from 5.4 ± 2.9% to 25.3 ± 8.6% (Fig. 3a). Even more striking, in addition to conferring an M2-like molecular phenotype, M1 macrophages cultured in the presence of QA gained the ability to suppress T-cell proliferation (from 76.8 ± 10.3% to 54.2 ± 2.2%; Fig. 3b), thereby, functionally mimicking M2 macrophages. As a key function of M1 macrophages is phagocytosis, we went on to determine if QA-induced skewing of macrophage polarization could result in a functional consequence on phagocytosis efficiency. As expected, murine derived microglia polarized towards the M1 phenotype demonstrated the unique ability of phagocytosis when compared to cells polarized towards the M2 phenotype, as measured by fluorescently labeled β-amyloid peptide (Fig. 3c; 92.2% vs. 18.7%). Intriguingly, consistent with molecular studies, QA conferred an M2-like phenotype to M1 macrophages, significantly attenuating their phagocytosis efficiency (38.8%). Importantly, these findings in mouse lines were further validated in human microglia derived from induced pluripotent stem cells (iPSCs; iCell; Fig. 3d) and murine-derived M1 macrophages (Suppl. Fig. 8).

### QA modulates M2 macrophage polarization through the NMDAR/Foxo1/PPARγ signaling axis

**Fig. 4:**
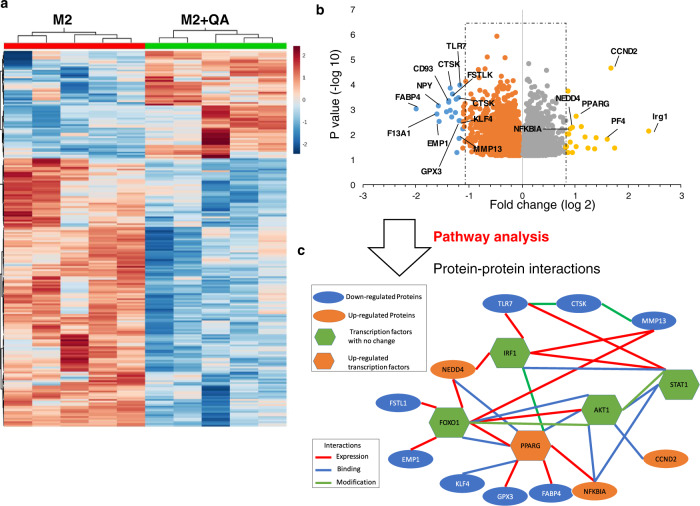
QA modulates PPARγ mediated transcriptional programs in M2 macrophages. M2 macrophages cultured in presence or absence of QA (20 µM) were flow-sorted for M2 macrophages (CD45 + CD11b + F4/80 + CD206 + ), and mRNA analyzed using Affymetrix arrays (Clariom™ D Assay, mouse; *n* = 5 biologically independent samples/group). **a** Heatmap demonstrates differentially expressed genes (log 1.5-fold change). **b** Differentially expressed genes (*p* < 0.05) are presented as a volcano plot. **c** Protein-protein interaction and pathway analysis. Statistics: Two-tailed Student’s *t*-test (**a**, **b**). All tests were performed at 95% confidence interval. Source data are provided as a source data file.

As QA’s upstream metabolic intermediary Kyn has been shown to modulate immune response through aryl hydrocarbon receptor (AhR) activation^12^, as an initial investigation, we sought to determine if this transcription factor served as a potential target and underlying mechanism of QA-induced modulation of macrophage plasticity. Utilizing cells transfected with a luciferase reporter functionally linked to the AhR-response promoter, we confirmed that Kyn, but not QA, has the unique ability to activate the AhR (Suppl. Fig. 9a). To provide a framework in identifying mechanistic underpinnings linking QA with M2 macrophage polarization, we extended previous studies to define global changes of QA-induced gene expression in M2 macrophages. In these studies, M2 macrophages (CD45 + CD11b + F4/80 + CD206 + ) cultured ±QA were flow-sorted and mRNA was isolated and profiled using an Affymetrix mouse array (Fig. 4a). To begin to understand the signaling machinery orchestrating QA-induced M2 macrophage polarization, Pathway Commons was used to map protein-to-protein interactions between the top 25 differentially expressed genes identified by volcano plot (Fig. 4b), identifying peroxisome proliferator-activated receptor gamma (PPARγ), which has been previously linked to M2 macrophage polarization^36,46^, as a lead candidate (Fig. 4c).

**Fig. 5:**
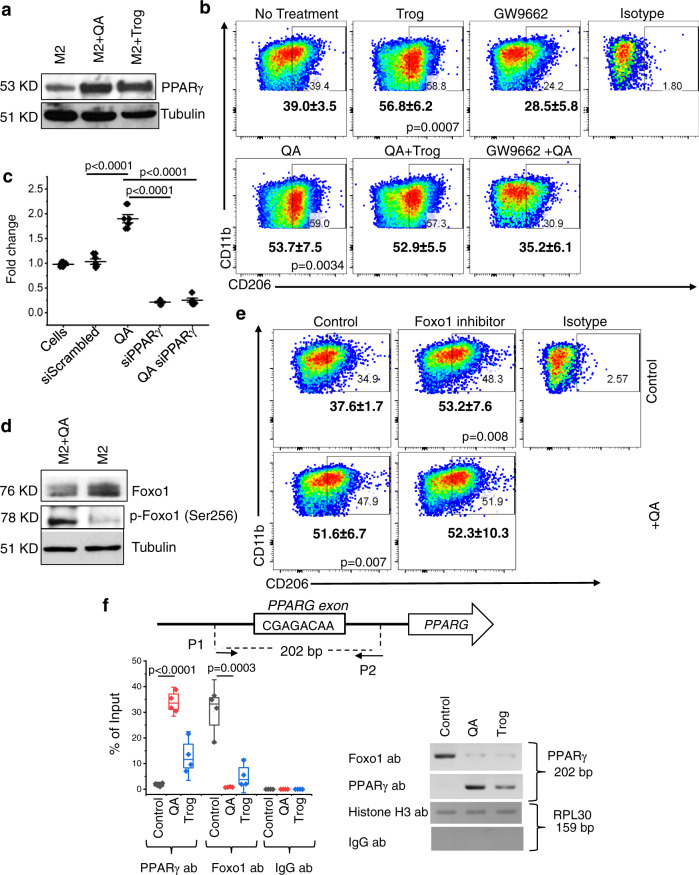
QA regulates PPARγ signaling using Foxo1. **a** Macrophages obtained from C57BL/6 mice were polarized towards the M2 phenotype ±QA (20 µM) or the PPARγ agonist troglitazone (Trog; 5 µM) and evaluated for the indicated proteins by western blot, which is representative of three biologically independent experiments. **b** Macrophages obtained from C57BL/6 mice were polarized towards the M2 phenotype ±Trog (*n* = 5), GW9662 (PPARγ antagonist; *n* = 3) and/or QA (20 µM; *n* = 5) and analyzed for M2 macrophage markers (CD45 + CD11b + F4/80 + CD206 + ) by flow cytometry. All samples were biologically independent. Numbers represent mean ± SD. **c** PPARγ transcriptional activity was evaluated in the murine macrophage cell line IC-21 polarized towards the M2 phenotype ±siRNA of indicated proteins by PPARγ transcriptional activity ELISA (*n* = 5 biologically independent samples). **d** C57BL/6 mouse-derived macrophages were polarized towards the M2 phenotype ±QA (20 µM) and evaluated for the indicated proteins by western blot, which is representative of 3 biologically independent experiments. **e** Macrophages were cultured in the presence or absence of QA ± the Foxo1 inhibitor AS-1842856 (0.1 µM) and evaluated for M2 macrophage markers (CD45 + CD11b + F4/80 + CD206 + ) by flow cytometry. Numbers represent mean ± SD (*n* = 4 biologically independent samples/group). **f** Chromatin immunoprecipitation (ChIP) was performed on C57BL/6 mouse-derived macrophages polarized towards the M2 phenotype and evaluated alone (gray), with QA (red) or with Trog (blue). ChIP was performed using antibodies against PPARγ, Foxo1, and histone 3 (positive control), and IgG antibody (negative control). After IP and DNA purification, the PPARγ exon (202 bp in length) was analyzed using qPCR and gel electrophoresis. Box plots represent interquartile range, line between the data points represents mean, and whiskers represents SE (*n* = 4 biologically independent samples/group). Statistics: One-way ANOVA followed by Tukey’s multiple comparisons test (**b**, **c**), two-tailed Student’s *t*-test (**e**, **f**). All tests were performed at 95% confidence interval. Source data are provided as a source data file.

To extend transcriptomic findings, we evaluated for differential expression of PPARγ in our models. Consistent with microarray findings, M2 macrophages cultured in the presence of QA demonstrated increased expression of PPARγ (Fig. 5a). As expected, culturing M2 macrophages in the presence of the PPARγ agonist troglitazone demonstrated a similar increase in PPARγ expression, and importantly, the combination of QA and troglitazone did not lead to any further increase in expression (Suppl. Fig. 9b), suggesting a commonality in underlying mechanisms. Next, we extended these findings to evaluate for M2 macrophage polarization. Consistent with western blot data, troglitazone increased CD206 + macrophages in a similar manner as QA, however, no further increases were observed with the combination, again supporting a commonality in underlying mechanisms (Fig. 5b). To further validate this pathway, M2 macrophages were polarized in the presence of the PPARγ antagonist GW9662. As expected, this led to decreased levels of CD206 + macrophages and was no longer influenced by QA, further supporting the hypothesis that QA modulates M2 macrophage polarization through PPARγ, which we went on to molecularly validate in the mouse macrophage IC-21 line using siRNA against PPARγ (Suppl. Fig. 9c). Lastly, to directly determine if QA activates PPARγ-dependent transcriptional programs in M2 macrophages, we utilized a PPARγ specific transcriptional activation assay, further supporting the role QA plays in modulating PPARγ transcriptional activation (Fig. 5c).

We next sought to determine how QA leads to PPARγ activation. Re-analyzing transcriptional profiles generated from QA-induced M2 macrophages and specifically evaluating for known regulators of PPARγ, the transcriptional factor forkhead box protein O1 (Foxo1) emerged as a putative molecular mediator. The Foxo1 transcription factor plays key roles in several cellular processes, including proliferation, metabolism, and survival in various immune cells. Several studies have demonstrated that Foxo1 inhibits the activity of PPARγ by binding to its promoter and inhibiting transcription^47,48^, or by directly binding to PPARγ protein in a transrepressional manner, resulting in the inability of PPARγ to bind to its primary locus, the PPARγ response element (PPRE)^49^. Transrepressional activity is blunted by phosphorylation of Foxo1, inducing cytosolic localization and ubiquitination, leading to PPARγ activation^48^. We therefore explored the interface between QA and Foxo1 in further detail. Consistent with our working model, CD206 + macrophages cultured in the presence of QA led to diminished expression of the negative regulator of PPARγ, Foxo1, and increased phosphorylation at Ser256, signaling ubiquitination of this protein (Fig. 5d). These molecular findings were further validated using the Foxo1 inhibitor AS1842856, which increased M2 macrophage polarization in a manner similar to PPARγ activation and was no longer influenced by QA (Fig. 5e). Lastly, chromatin immunoprecipitation (ChIP) was performed, definitively establishing that QA attenuates Fox01 binding to the PPARγ promotor in a manner similar to the PPARγ agonist troglitazone (Fig. 5f). Interestingly, these studies uncovered a strong QA-induced positive feedback loop involving PPARγ expression and activation of its own promoter.

**Fig. 6:**
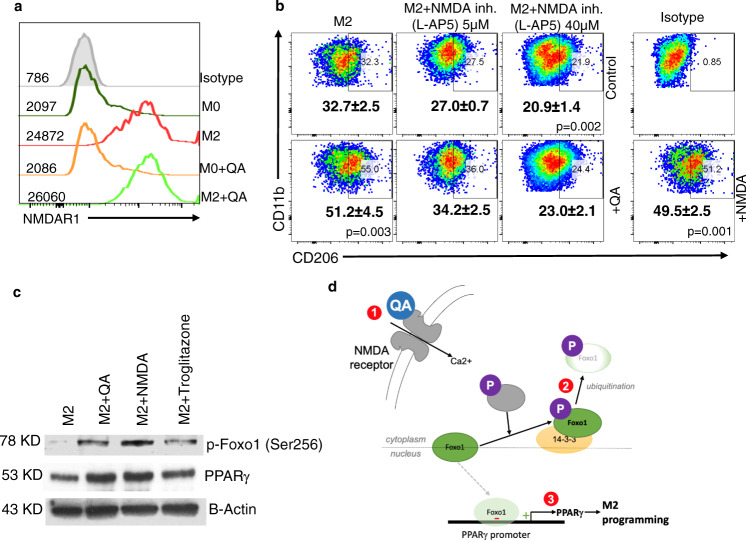
QA modulates M2 macrophage polarization through the NMDAR/Foxo1/PPARγ signaling axis. **a** M2 macrophages were polarized ±QA, gated for macrophage markers (CD45 + CD11b + F4/80 + ) and CD206 + ve with (green) or without (red) QA or CD206-ve with (orange) or without (dark green) QA, and evaluated for expression of the NMDA receptor 1 (NMDAR1; *n* = 2 biologically independent samples/group). **b** M2 macrophages were cultured in ±QA, L-AP5 (NMDAR1 inhibitor), or NMDA and cells were gated for M2 macrophages markers (CD45 + CD11b + F4/80 + CD206 + ). Numbers represent mean ± SD (*n* = 3 biologically independent samples/group). **c** M2 macrophages were cultured in ±QA, NMDA, or Trog, and indicated proteins were evaluated by western blot, which is representative of three biologically independent experiments. **d** Schematic depicting the proposed mechanism of QA-induced M2 macrophage polarization. QA binds to the NMDA receptor of macrophages (1), leading to phosphorylation of Foxo1 (2). Phosphorylated Foxo1 is retained in the cytoplasm and destined for ubiquitination. Loss of nuclear Foxo1, a negative regulator of the PPARγ promoter, leads to increased PPARγ expression and transcriptional programs designed to promote macrophage polarization towards the M2 phenotype (3). Statistics: Two-tailed Student’s *t* test at 95% confidence interval (**b**). Source data are provided as a source data file.

As we have established that QA modulates M2 macrophage polarization through the Foxo1/PPARγ axis, we next sought to determine the primary target of QA. As QA is a known ligand of the NMDA receptor, as an initial investigation, we evaluated for the expression of the NMDA receptor in macrophages. Interestingly, NMDAR1 expression (a key receptor subunit of this heterotetramer) was only observed in M2 macrophages but not unpolarized macrophages (Fig. 6a). Similar to QA, an increase in M2 macrophage polarization was observed when cultured in the presence of NMDA and consistent with our working model, the NMDAR competitive inhibitor L-AP5 demonstrated a dose-dependent inhibition of M2 macrophage polarization and mitigated the activity of QA (Fig. 6b). Lastly, we demonstrated that NMDA led to Foxo1 phosphorylation and increased PPARγ expression in a manner similar to QA and troglitazone (Fig. 6c). Collectively, we demonstrate QA-induced M2 macrophage polarization is mediated through the NMDAR/Foxo1/PPARγ signaling axis (Fig. 6d).

### Targeting the downstream metabolism of tryptophan in GBM by inhibiting KYNU

**Fig. 7:**
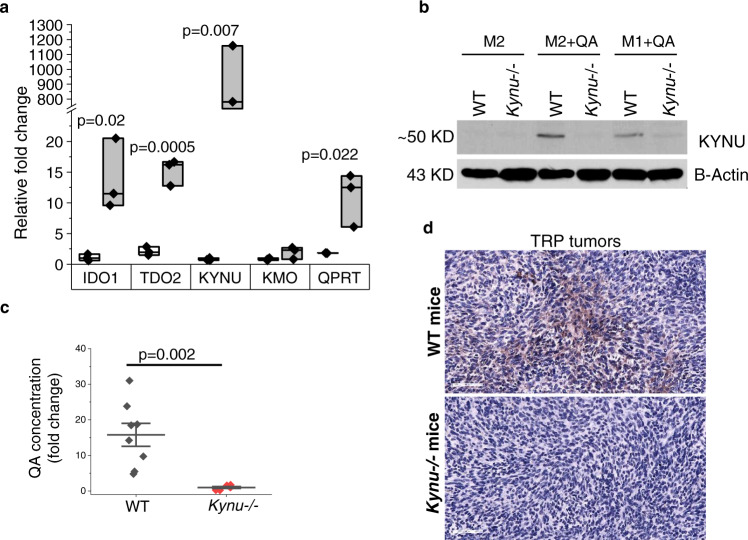
Targeting the downstream metabolism of tryptophan in GBM using the *Kynu*-/- model. **a** Macrophages obtained from C57BL/6 mice were polarized towards the M2 phenotype ±QA (20 µM), and RNA was isolated and evaluated for indicated genes using real-time PCR. Bar graph represent mean ± SD (*n* = 3 biologically independent samples/group). **b** Macrophages obtained from C57BL/6NJ *Kynu*-/- and WT mice were polarized towards the M1 or M2 phenotype in ±QA, and indicated proteins were evaluated by western blot, which is representative of three biologically independent experiments. **c**, **d** TRP tumors were grown orthotopically in C57BL/6NJ WT (*n* = 8; gray) or *Kynu*-/- (*n* = 6; red) mice and QA levels was determined by (**c**) ELISA and (**d**) immunohistochemical staining. All samples were biologically independent. Line between data points represents mean, and whiskers represent SE (**c**). Statistics: Two-tailed Student’s *t*-test (**a** and **c**). All tests were performed at 95% confidence interval. Source data are provided as a source data file.

The above findings support targeting the downstream metabolism of tryptophan may serve as a therapeutic strategy to overcome macrophage-induced immune suppression in GBM. As the primary cell able to generate QA from tumor-produced Kyn are macrophages, we evaluated for the expression of enzymes involved in the downstream metabolism of tryptophan in mouse derived macrophages using RT-PCR. Baseline levels of KMO, kynureninase (KYNU), and 3-HAO (3-hydroxyanthranilate 3,4-dioxygenase) appeared equivalent. However, interestingly, when cultured in the presence of QA, there appeared to be a strong positive feedback loop in all enzymes, but most notable in KYNU, which demonstrated a several log fold increase in expression (Fig. 7a). Based on these findings, KYNU emerged as our primary therapeutic target to inhibit QA accumulation in GBM. As agents effectively targeting this enzyme are not commercially available, we generated a *Kynu*-/- GEMM to allow us to extend these promising findings in vivo. C57BL/6NJ-Kynuem1J/Mmjax mice were developed using CRISPR-Cas9 knockout of *Kynu* exon 3 and 219 bp flanking intronic sequences and F3 (homozygous to homozygous mating). Successful knockout was validated using qPCR (Suppl. Fig. 10a). No clear phenotypic changes were observed in *Kynu*-/- mice (Suppl. Fig. 10b). We validated KYNU expression was abrogated in both M2 and M1 macrophages derived from *Kynu*-/- mice (along with the capacity of QA in induce expression of KYNU in macrophages from WT mice) by western blot (Fig. 7b) and the inability of the upstream metabolite Kyn to modulate polarization of these cells (Suppl. Fig. 10c). Next, we sought to validate cells contributing towards immune response remained intact in the *Kynu*-/- model. CD4 + T cells, CD8 + T cells, Tregs, B cells and NK cells generated from splenocytes of WT and *Kynu*-/- mice were quantitatively similar (Suppl. Fig. 10d) and CD4 + and CD8 + T cells demonstrated similar phenotypes upon anti-CD3/anti-CD28 activation, including proliferation (Ki67), TNFα and IFNγ inflammatory cytokine production, and T cell activation marker CD69 (Suppl. Fig. 10e). Further QA, but not Kyn, retained the ability to induce functional suppression in M2 macrophages derived from *Kynu*-/- mice (Suppl. Fig. 10f).

**Fig. 8:**
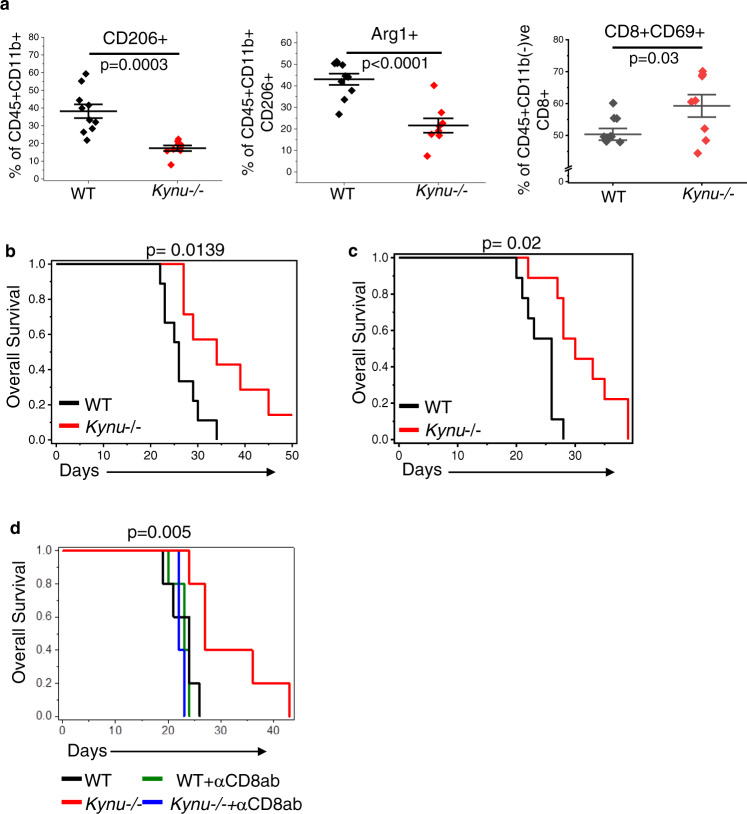
Targeting QA accumulation using the *Kynu*-/- model modulates the immune landscape and demonstrates anti-tumor activity in GBM in vivo. **a** TRP murine GBM tumors were grown orthotopically in C57BL/6NJ WT (*n* = 10; black) or *Kynu*-/- mice (*n* = 8; red). Mice were euthanized on day 21 and tumors harvested for immune profiling using flow cytometry, including M2 macrophages (CD45 + CD11b + F4/801 + CD206 + ); Arginase 1 + M2 macrophages (CD45 + CD11b + F4/801 + CD206 + Arg1 + ), and activated CD8 + T cells (CD45 + CD8 + CD69 + ). Line between the data points represents mean and whisker represents SE. All samples were biologically independent. **b** TRP murine GBM tumors were grown orthotopically in C57BL/6NJ WT (black; *n* = 9/group) or *Kynu*-/- (red; *n* = 7/group) mice and followed for survival using a Kaplan–Meier survival plot. **c** GL261 murine GBM tumors were grown orthotopically in C57BL/6NJ WT (black) or *Kynu*-/- (red) mice and followed for survival using a Kaplan–Meier survival plot (*n* = 9/group). **d** TRP murine GBM tumors were grown orthotopically in C57BL/6NJ WT without (black) and with (green) CD8 T-cell depletion and *Kynu*-/- mice without (red) and with (blue) CD8 T-cell depletion using an anti-CD8 antibody delivered i.p. weekly (*n* = 5/group). All samples were biologically independent. Statistics: Two-tailed Student’s *t*-test (**a**), log rank *p*-value (**b**, **c** and **d**). All tests were performed at 95% confidence interval. Source data are provided as a source data file.

Next, using our *Kynu*-/- mouse model, we examined the influence of QA on the immune landscape of GBM in vivo and its potential to serve as a therapeutic target. As an initial investigation, using the mouse GBM cell line TRP^10,33,34^, tumors were grown intracranially in WT and *Kynu*-/- mice, sacrificed on day 25, and tumors harvested for correlative studies. As expected, QA was nearly undetectable in tumors grown in *Kynu*-/- mice when evaluated by both ELISA (Fig. 7c) and IHC (Fig. 7d). No change in lymphocytes were observed when comparing WT and *Kynu*-/- mice; however, consistent with in vitro studies, a significant decrease in total macrophages was observed in tumors grown in *Kynu*-/- mice (Suppl. Fig. 11a). Further, as our in vitro models predicted, a significant decrease in both CD206 + macrophages and M2 macrophages expressing the suppressive marker Arginase 1 was observed in GBM grown in *Kynu*-/- mice devoid of QA (Fig. 8a). As expected, no changes in MDSCs or Tregs were observed (Suppl. Fig. 11b). Although no changes in numbers of CD4 + or CD8 + cells were observed, tumors grown in *Kynu*-/- mice had higher levels of CD8 + CD69 + T cells (Fig. 8a), suggesting immune activation. Lastly, we performed imaging and survival studies to determine the potential of KYNU to serve as a therapeutic target. MR imaging performed on day 21 demonstrated a significant decrease in volume of tumors grown in *Kynu*-/- mice (Suppl. Fig. 11c). *Kynu*-/- tumor bearing mice demonstrated a significant improvement in median (25 days vs. 35 days) and overall survival, including ~50% of these mice still being alive when the last WT mice were euthanized, with several achieving long-term survival (Fig. 8b). These findings were validated when we extended this line of investigation to the mouse GBM line GL261, including both an improvement in survival observed in *Kynu*-/- tumor bearing mice (Fig. 8c) and the influence of QA on the immune suppressive microenvironment (Suppl. Fig. 12).

Lastly, to definitively determine if the anti-tumor activity observed in *Kynu*-/- mice was a direct consequence of QAs role in immune suppression, we performed CD8 + T cell depletion studies. Correlative studies validated CD8 + depletion and previously described decreases in CD206 + and Arg1+ macrophages in *Kynu*-/- mice (Suppl. Fig. 13). CD8 depletion completely mitigated the anti-tumor activity observed in *Kynu*-/- mice, providing strong support for QAs immune suppressive role in GBM and its therapeutic potential (Fig. 8d). Collectively, these studies support the contributory role of QA in sculpting the immune suppressive microenvironment in GBM and the potential for this pathway to serve as a therapeutic target.

## Discussion

GBM continues to be an invariably fatal malignancy with limited treatment options. Despite continued advancements in surgical techniques, accuracy of radiation therapy delivery, and systemic therapy, median overall survival (OS) remains <2 years^50^. Immune checkpoint agents represent a powerful tool in the armamentarium of available therapies, revolutionizing how cancer is treated. Unfortunately, clinical gains demonstrated in a majority of other tumor types has not translated to improved outcomes in GBM patients^51–55^. One likely reason underlying this lack of clinical benefit of immune checkpoint inhibitors in GBM is the primary target of these agents may not play a dominant role in this tumor’s immune suppressive microenvironment. Rather than efforts designed to re-activate T cell antitumor activity by blocking the programmed cell death ligand-1 (PD-L1) axis or cytotoxic T-lymphocyte-associated protein 4 (CTLA-4), recent evidence suggests that TAMs play a more dominate role in the observed immunosuppression of GBM^3,32^. Macrophages represent a dynamic population of immune cells whose phenotype represents a heterogenous continuum from tumor-supportive (M2-like) and pro-inflammatory, tumor-suppressive (M1-like). Although we acknowledge that the M1/M2 dichotomy represents an oversimplification, it does provide a framework to begin to understand the interface between tumors and their immune-microenvironment. This is particularly relevant in the context of GBM, where M2-like TAMs are found in higher percentages in their microenvironments compared to other tumor types, suggesting they play a contributory role in the immune suppressive microenvironment found in these tumors^32,56^. Hence, there is considerable interest in developing therapeutic strategies designed to either inhibit the accumulation of tumor supportive macrophages or revert their phenotype to enhance anti-tumor immunity in GBM. In these studies, we demonstrate the downstream metabolism of tryptophan, resulting in the accumulation of QA, represents a metabolic phenotype of GBM and show how this metabolic pathway sculpts the immune microenvironment by influencing macrophage polarization. As a major emphasis in the field has been the upstream metabolism of this pathway, namely, inhibiting the accumulation of the immune modulating metabolite Kyn by targeting IDO1 and/or TDO, these findings provide a window into the underlying biologic relevance of this pathway and its therapeutic implications. Specific to GBM, although increased expression of upstream enzymes IDO and TDO have been observed, along with the accumulation of Kyn^12,57^, targeting these pathways individually did not result in notable changes in the immune profile of these tumors or independent anti-tumor activity in preclinical models^10,13^, and unfortunately, as these investigations predicted, did not result in clinical benefit^58,59^. Based on our findings, targeting the downstream metabolism of tryptophan may have more relevance to the immune landscape of GBM and it is tempting to speculate, have a similar immune modulatory role in other types of cancers. Specifically, polarizing macrophages in the presence of QA conferred an M2-like molecular phenotype, including nearly a 50% increase in expression of the M2 macrophage marker CD206 and the immune-suppressive markers IL4Rα + and Arginase 1. Importantly, in addition to these molecular markers, which were further extended through transcriptional profiling, QA conferred potent suppressive properties to these cells. Unexpectedly, QA also conferred an immune-suppressive phenotype to macrophages polarized in conditions promoting M1 differentiation. Specifically, in addition to expressing the M2 macrophage marker CD206, these cells functionally recapitulated M2 macrophages, having both suppressive properties and diminished phagocytosis capacity when tested using β-amyloid peptide. This offers the intriguing possibility that in addition to cancer, targeting this immune modulatory metabolite may have therapeutic implications in Alzheimer’s disease, which represents one of several neurodegenerative diseases where an accumulation of QA has been observed^19^. Acknowledging the oversimplification of utilizing an M1/M2 dichotomy, future studies are designed to incorporate single-cell sequencing to better define specific macrophage subpopulations influenced by QA.

Through global gene expression profiling, in addition to discovering a role the NMDA receptor plays in macrophage polarization, we uncovered a mechanism linking QA-induced receptor activation to established pathways involved with M2 macrophage polarization. Specifically, through both chemical and molecular perturbation of this pathway coupled with ChIP analysis, we demonstrate QA leads to phosphorylation and degradation of the transcription factor Foxo1, which serves as an endogenous inhibitor of PPARγ expression through promoter binding. This resulted in both increased PPARγ expression, its transcriptional activation, and the observed increased M2 macrophage polarization. Further studies are warranted to more comprehensively establish the role of NMDA receptor activation and macrophage polarization, how this may apply to different pathophysiological states, and its therapeutic potential.

We went on to extend data suggesting QA contributes towards the immune suppressive microenvironment of GBM by skewing macrophages towards a tumor supportive state in vivo. We observed a potent positive feedback loop involving KYNU expression in macrophages cultured in the presence of QA, making it our lead candidate to inhibit the downstream metabolism of tryptophan. As agents are currently not commercially available to inhibit this enzyme, we generated a *Kynu*-/- knockout mouse model. These mice were phenotypically similar to wild-type mice and had identical immune profiles. Consistent with in vitro findings, tumors grown orthotopically in *Kynu*-/- mice demonstrated a dramatic reduction in M2-like macrophages, and importantly, this was coupled with an increase in immune activation. Tumors implanted in *Kynu*-/- mice demonstrated decreased growth, resulting in improved survival, including several mice achieving long-term survival in two mouse-derived GBM cell lines. To definitively determine if the anti-tumor activity observed in *Kynu*-/- mice was a direct consequence of QAs role in immune suppression, CD8 + T cell depletion studies were performed, which completely mitigated the anti-tumor activity observed in *Kynu*-/- mice, providing further support for QAs immune suppressive role in GBM and its therapeutic potential. Perhaps more significant than the independent anti-tumor activity observed by targeting QA accumulation in GBM, the resulting reduction of the immune suppressive microenvironment offers exciting opportunities to enhance current therapeutic strategies reliant on immune activation, including CAR T-cell therapy, GBM vaccines, and established immune checkpoint inhibitors. Studies are ongoing designed to test these promising combinatorial approaches. As the link between tryptophan metabolism and cancer is well recognized^13,60^, and both preclinical and clinical studies designed to target the upstream metabolism of this pathway have thus far been disappointing^59^, these findings offer an opportunity to both revisit the biologic relevance of this pathway as it relates to oncogenesis. In addition, it provides the framework to begin to identify compounds that can selectively target the downstream metabolism of tryptophan, thereby serving as a panel of immune modulatory agents that may be applied in GBM, others cancers, and neurodegenerative diseases where this pathway has been implicated.

## Methods

### Study approval

All studies were performed under protocols approved by the Institutional Animal Care and Use Committee at William Beaumont Research Institute (AAALAC-accredited facility).

### Human tumor samples and metabolomic/genomic profiling

These data were not generated for the purpose of this study and obtained from ref. ^28^. Metabolomic profiling, gene expression profiling, GBM subtyping, O6-methylguanine-DNA methyltransferase (MGMT) promoter methylation, and isocitrate dehydrogenase 1 (IDH1) mutation analyses of patient-derived tumors were performed as previously described^10,28^.

### Cell culture and reagents

The TRP GBM line was generated from genetically engineered mice and cultured in MEM as previously described^10,33,34^. Briefly, primary astrocyte lines were generated from a series of conditional GEM in which core GBM pathways were genetically targeted. Cre-mediated recombination in vitro lines express (1) a truncation mutant of SV40 large T antigen (T) from the human Gfap promoter that inactivates all 3 Rb family proteins, (2) a constitutively active KrasG12D mutant (R), and/or (3) a homozygous Pten deletion (P). GL261 cells (murine GBM) were purchased from NIH’s DCTD tumor repository (#GL261) and cultured in DMEM. IC-21 cells (murine macrophage) were purchased from ATCC (#TIB186) and cultured in RPMI-1640. Human iPSC-derived microglia cells were purchased from Fujifilm Cellular Dynamics, Inc. (Madison, WI; #C1110) and cultured as described by Abud et al.^61^. Briefly, cells were cultured in DMEM/F-12 media with N-2, B27, glutamax, ITS-G, and human insulin supplements (GIBCO/Thermo, Grand Island, NY) along with 1-thioglycerol, ascorbic acid and MEM non-essential amino acids (Millipore/Sigma, St. Louis, MO). Human iPSC-derived microglia exhibit functional characteristics like human microglia, including phagocytosis and cytokine-mediated inflammatory responses, and express relevant microglial markers (TREM2, CX3CR1, TMEM119, P2RY12, IBA1). These markers and characteristics were verified by the vendor. In addition, cells were cultured in the presence of cytokines rhM-CSF (25 ng/ml), rhTGF-β1 (50 ng/ml), rhIL-34 (100 ng/ml), rhFractalkine (100 ng/ml) (PeproTech, Rocky Hill, NJ) and 100 ng/ml of human CD200 (Acro Biosystems, Newark, DE). On day five, rhIFN-γ (50 ng/ml) and LPS (50 ng/ml) were added to polarize these cells towards the M1 phenotype. Primary cell cultures of mouse splenocytes (T cells) was performed in RPMI-1640. All cell culture reagents were purchased from GIBCO/Thermo (Grand Island, NY) and Corning Life Sciences (New York, NY). Tryptophan, Kyn, QA, NAD + , and LPS (E. Coli O111:B4) were purchased from Sigma Aldrich (St. Louis, MO). All cytokines for murine macrophage polarization (mIL-4, mIL-13, mIFN-γ, hIFN-γ, hTGF-β, hIL-2, and mGM-CSF) were purchased from PeproTech (Rocky Hill, NJ). KMO inhibitor (UPF648), PPARγ inhibitor (GW9662), Foxo1 inhibitor (AS-1842856), PPARγ agonist (troglitazone), were purchased from Cayman Chemicals (Ann Arbor, MI). NMDAR agonist (NMDA) and antagonist (L-AP5) were purchased from Tocris Bioscience (Minneapolis, MN). GDC-0919 was provided by Genentech (San Francisco, CA). Antibodies were purchased from BioLegend (San Diego, CA) or eBioscience/Thermo (Grand Island, NY). Clones for antibodies used for flow cytometry are as indicated: CD45 (30-F11), F4/80 (BM8), CD11b (M1/70), CD206 (C068C2), Arg 1 (A1exF5), IL4Ra (JAMA-147), CD80 (16-10A1), Gr1 (RB6-8C5), FoxP3 (150D/E4), CD8 (53-6.7), CD4 (Gk1.5), CD25 (PC61), CD69 (H1.2F3), GzmB (QA16A02) and Tmem119 (V3RT1G0sz). Matched isotype mAb and mouse Fc block CD16/CD32 antibody was obtained from BioLegend (San Diego, CA). Fluorochrome-linked anti-NMDAR1 antibody (N308/48) was obtained from Abcam (Waltham, MA). CD8 T cell depletion antibodies (clone: 2.43) were purchased from Bio X Cell (Lebanon, NH). A detailed summary of all antibodies used in this study is provided in Suppl. Table 1.

### Quinolinic acid quantification

QA was quantified using ELISA (Abclonal, Woburn, MA). Tryptophan (Trp), QA, and KYN were analyzed using or gas-chromatography–mass spectrometry^62,63^. Briefly, weighted tumor tissues or an equal number of pelleted cells were treated with equal parts of ice-cold methanol and water. Subsequently, the cell pellet was homogenized. Ice-cold chloroform was added to the cell pellets, followed by centrifugation to get a phase separation. The aqueous phase was air-dried and derivatized (75 °C) using PFP (2,2,3,3,3-pentafluoro-1-propanol) and PFPA (pentafluoropropionic anhydride), and the derivatized samples were subsequently dried using a stream of nitrogen. Dried samples were dissolved in ethyl acetate and acquired using Agilent 7000 C triple quad mass spectrometer and Agilent 7890 GC (Agilent, Santa Clara, CA). 1 µL sample was injected using Agilent 7693 auto-sampler injector operated in electron capture negative ionization (ECNI) mode. Trp, QA and KYN were quantified by selected ion monitoring of QA (431 m/z), KYN (454 m/z), and Trp (608 m/z). Data were analyzed using MassHunter version B (Agilent, Santa Clara, CA). Similar cell pallets or tissues were spiked with different concentrations of QA and compared with test samples to get an approximate quantification of QA. To analyze tryptophan flux between tumor cells and macrophages we used the isotope of L-tryptophan (L-Tryptophan-2,3,3-d3 207 gm/mol) instead of L-tryptophan (204 gm/mol) in cell cultures. L-Tryptophan-2,3,3-d3 was obtained from CDN Isotopes (Quebec, Canada). Trp, QA and KYN were quantified by selected ion monitoring of QA (432 m/z), KYN (456 m/z), and Trp (610 m/z).

### Flow cytometry

Cells were washed with FACS buffer (PBS supplemented with 0.2% BSA, 0.01% NaN3) and stained with fluorochrome-conjugated-conjugated mAbs along with mouse Fc receptor block (clone: 93). Fixation and permeabilization was performed using a eBioscience/Thermo kit. All samples were analyzed on a FACS Canto II flow cytometer (Becton Dickinson; Mountain View, CA). Analysis of flow cytometry data was performed using FlowJo V10 software (FlowJo, LLC; Ashland, OR). Data was represented as two parameter flow cytometry plots in log scale, with biexponential transformation. Our positive gating strategy uses isotype controls to help us differentiate non-specific background signals from specific antibody signals. A summary of FACS gating/sorting strategies are provided in Suppl. Fig. 14.

### Immune cell polarization and characterization

#### Macrophage polarization

Bone marrow cells were isolated from the indicated mouse line and cultured in GM-CSF (40 ng/ml) for 5 –7 days to generate M0 cells. Cells were then cultured with IL-4 (20 ng/ml) and IL-13 (20 ng/ml) for M2 polarization or IFN-γ (50 ng/ml) and LPS (100 ng/ml) for M1 polarization (2 d). M1 or M2 macrophages were stained with fluorochrome-conjugated mAbs against CD45, F4/80, CD11b, CD80, CD206 and/or iNOS. Total macrophages were characterized using CD45 + CD11b + F4/80+ cells. These macrophages were further categorized into M1-like macrophages (CD45 + CD11b + F4/80 + CD206-veCD80^high^iNOS + ) based on presence of CD80 or iNOS and absence of CD206. Similarly, CD206 + CD80^low^ cells were considered as M2-like macrophages (CD45 + CD11b + F4/80 + CD206 + veCD80^low^). Arginase 1, IL-4Rα, and CD209 (M2 specific macrophage markers) were also analyzed in M2 polarized macrophages. *MDSC polarization:* Bone marrow cells were cultured for 5 days in GM-CSF (40 ng/ml) and IL-6 (40 ng/ml) to generate MDSCs. MDSCs were characterized using CD45 + CD11b + Gr1+ markers. *Microglial cell polarization:* Microglial cells were isolated from the healthy brains of C57BL/6 mice using the Percoll density gradient centrifugation method^64^. Isolated microglial cells were confirmed for CD45 low expression and CD11b + TMEM119 + staining. Microglial cells were cultured in GM-CSF (40 ng/ml) for 5 days to generate M0-MDMs (microglial-derived macrophages). Cells were then cultured with IL-4 (20 ng/ml) and IL-13 (20 ng/ml) for M2 -like MDMs (MDM2s) polarization or IFN-γ (50 ng/ml) and LPS (50 ng/ml) for M1-MDMs polarization (2 d). M1-like MDMs (MDM1s) were characterized using CD45^low^CD11b + F4/80 + CD206-veCD80^high^ markers. Similarly, MDMs were characterized using CD45l^ow^CD11b + F4/80 + CD206 + veCD80^low^ markers. *iTreg polarization:* Magnetic bead sorted naïve CD4 + T cells were isolated from C57BL/6 mice splenocytes. CD4^+^ T cells were polarized to CD4^+^ FoxP3^+^ using rhTGF-β (30 ng/ml) and rhIL2 (300 IU/ml) and activated using plate-bound anti-CD3 (1 µg/ml; clone-145-2C11) and anti-CD28 (5 µg/ml; clone- 37.51) antibodies (Biolegend; San Diego, CA) for 4 days. CD4 + CD25 + and FoxP3+ cells were considered as iTregs.

### T cell culture and activation

Primary splenocytes were obtained from C57BL/6NJ or C57BL/6NJ-*Kynu*-/- mice. Naïve T cells (CD4 + and CD8 + T cells) were sorted from splenocytes using Dynabeads untouched magnetic sorting kit (Grand Island, NY). Purified CD4 + and CD8 + T cells were activated with plate-bound anti-CD3 (1 µg/ml; clone- 145-2C11) and anti-CD28 (5 µg/ml; clone- 37.51) antibodies (Biolegend) for 3 days. CD4 + and CD8 + T cells were analyzed for markers of activation (CD69 + ) and proliferation (Ki67). Activated CD4 + and CD8 + T cells were stimulated with a cell activation cocktail PMA/Ionomycin with Brefeldin A (Biolegend) overnight and stained for IFN-γ and TNF-α after fixation and permeabilization using the eBioscience/Thermo kit (Grand Island, NY). Cells were and acquired using the FACS Canto II flow cytometer.

### CD8 T cell proliferation/suppression

CD8^+^ T lymphocytes were sorted using a Dynabead FlowComp Mouse CD8 Kit (Grand Island, NY). Cells were stained with CFSE CellTrace (GIBCO/Thermo; Grand Island, NY) and activated with plate-bound anti-CD3 (1 µg/ml; clone- 145-2C11) and anti-CD28 (5 µg/ml; clone- 37.51) antibodies (Biolegend). For suppressing proliferation, polarized M2 macrophages were added at varying concentrations with and without Kyn or QA. CFSE dilution on CD8^+^ T cells was analyzed 3 days after activation using a FACS Canto II flow cytometer (Becton Dickinson, Mountain View, CA). CFSE dilution was analyzed on CD8^+^ T cells after 3 days of activation. CFSE stained cells were also stained for Granzyme B, (clone 29 F.1A12).

### Phagocytic ability

For analysis of the phagocytic ability of cells, we used green (HiLyte Fluor 488) florescent β-amyloid (1–42) peptide (Anaspec, Fremont, CA). Macrophages/microglial cells were pulsed with 400 nM of florescent peptide for 16 h, followed by washing and removal of the soluble florescent probe. Cells were then stained for surface markers (CD45, F4/80, CD11b, CD206, or CD80) and analyzed for green florescence after gating appropriate macrophage subset.

### Western blot

Western blot was performed using methods previously described^10^. PPARγ (C26H12), Foxo1 (C29H4), pFoxo1 (Ser256) (#9461), and tubulin (#2144) antibodies were obtained from Cell Signaling Technology. Mouse KYNU (polyclonal), tubulin, and actin antibodies were obtained from Thermo Fisher.

### Real-time qPCR

Total RNA was extracted from pellets of polarized macrophages, and mRNA was extracted using RNA mini kit (BioRad, Hercules, CA), and cDNA was generated from total RNA using iScript cDNA Synthesis Kit (BioRad, Hercules, CA). SYBR Green (Biorad, Hercules, CA) was used for quantitative PCR using Vii7 real-time PCR (Grand Island, NY). Primers were synthesized using a predesigned qPCR primer from IDT (Coralville, IA). Expression mRNA was normalized to ACTB and HPRT.

### Murine microarray and data analysis

Murine M2 polarized cells were gated for CD45 + CD11b + F4/80 + CD206 + and flow-sorted using FACS Aria II (Becton Dickinson; Mountain View, CA). Cells were >98% pure. Total RNA was isolated from these cells using the Qiagen all prep kit (Hilden, Germany; cat # 80204) and quantified using a Nanodrop. Integrity of RNA preparation was confirmed with the Agilent RNA 6000 Nano Kit (cat # 5067-1511). 500 ng of total RNA was used for Clariom D mouse Transcriptome Array 1.0 (MTA 1.0.). Raw data were processed using the Affymetrix expression console, and further data analyses were performed using the Affymetrix transcriptome analysis console. Raw data were normalized using gene-level RMA Sketch, and bi-weight average signal (log2) values were used for analysis. To determine the association between various mRNA gene expression, we performed hierarchical clustering with log2 transformed normalized data using Euclidean distance and Ward scaling using MetaboAnalyst 5.0^65^. Fold change was calculated and used in generating the volcano plot (log2 fold change on the *x*-axis and negative log10 of *p*-value on the *y*-axis). Using the top 24 genes from the volcano plot, we performed protein-protein interaction analysis using Pathway Commons version 12^66^. Transcriptions factors (AP1, AP4, FOXO1, FOXO4, PPARγ, OCT1, AKT1, NFKB, STAT1, IRF1, IRF2, IRF7) associated with target genes were integrated into the analysis to identify interactive pathway.

To calculate the diverse activation states and phenotypes of macrophages, we performed a differential abundance score (DA score) analysis using microarray and data from flow-sorted M2 macrophages and M2 macrophages polarized in the presence of QA. Differentially expressed genes (Mann-Whitney U tests and Benjamini-Hochberg corrected *p*-value < 0.05) were used for this analysis. Each transcript was classified into major pathways based on the literature search. The DA score was calculated using the formula.

DA = (Number of transcripts significantly upregulated in M2 + QA—number of transcripts significantly downregulated in M2 + QA)/total number of transcripts in the pathway

A score of 1 indicates all genes in a pathway were increased, and a score of −1 indicates all the metabolites or genes in a pathway decreased. The plot was generated by computing DA score on the *x*-axis and the pathway on the *y*-axis. The size of the bubble represents the number of transcripts in the pathway^10,28^.

### Aryl hydrocarbon receptor (AhR) activity reporter assay

AhR activation was analyzed using AhR reporter cells (INDIGO Biosciences, PA) according to the manufacturer’s protocol.

### Chromatin immunoprecipitation (ChIP)

ChIP was performed using a ab500-ChIP kit (Abcam, Cambridge, MA) according to the manufacturer’s protocol. Briefly, cells were cross-linked in 1.1% formaldehyde and then re-suspended in cell lysis buffer followed by sonication for DNA fragmentation (200-1000 bp). Chromatin was sonicated and then incubated with a polyclonal antibody against PPARγ (81B8 and C26H12) or FoxO1 (C29H4) Cell Signaling Technology (Danvers, MA) for immunoprecipitation. Antibodies for histone H3 was used as a positive control and IgG as a negative control. ChIP-enriched DNA was further purified and quantified by real-time PCR for the PPARγ gene and normalized to input DNA control samples using PPARγ primers F-5'-CCACTGGTGTGTATTTTACTGC-3' and R-5'-AAAATGGTGTGTCATAATGCTG-3'^67^. RLP30 primers were obtained from Cell Signaling Technology and used as a control.

### PPARγ transcriptional activation

PPARγ Transcription Factor ELISA Assay was used to analyze PPARγ binding activity according to the manufacturer’s protocol (Cayman Chemicals, Ann Arbor, MI).

### PPARγ silencing

PPARγ silencer select (assay ID- s72013) and non-targeting (scrambled si; Thermo Fisher) was used for transient knockdown. IC-21 cells were cultured in ±QA for 6 days in RPMI-1640 along with GM-CSF (40 ng/ml). Cells were then transfected with siRNAs using Lipofectamine 2000 (Thermo Fisher Life Technologies) for 8 h in Opti-MEM media (Thermo Fisher). Cells were re-suspended in macrophage media containing IL4 and IL13 (20 ng/ml) for 24 h to polarize towards the M2 phenotype. Cells were then transfected with siRNAs using Lipofectamine 2000 (Thermo Fisher Life Technologies) for 8 h in Opti-MEM media (Thermo Fisher) and re-suspended in macrophage conditioning media.

### Animal studies

All animal studies were carried out under protocols approved by the Institutional Animal Care and Use Committee at William Beaumont Research Institute (AAALAC-accredited facility) under animal protocols AL-15-09, AL-18-10, AL-19-07, AL-21-04, and AL22-06. These protocols utilize strict criteria to require euthanization of mice, which includes demonstration of neurologic symptoms, weight loss, decreased activity, and other factors suggestive of illness. C57BL/6 mice were purchased from Charles River Laboratories (Wilmington, MA). Mice were fed a Teklad Global Rodent Diet (2020X; Envigo [Madison, WI]) ad libitum and housed in a vivarium with up to 5 mice/cage. C57BL/6NJ-*Kynu*-/+ and C57BL/6NJ were purchased from Jackson Labs (Bar Harbor, ME) and bred at the Beaumont Research Institute animal facility to generate C57BL/6NJ-*Kynu*-/- mice. Mice with *Kynu* gene deletion were identified by Taqman probe PCR using primers (Integrated DNA Technologies, Coralville, IA) for *Kynu* deletion (F) 5'-CCTTGGCTATTTGTGATTGG-3'; *Kynu* wild type (F) 5'-GAAATTCCCTTGGCCTTCA-3' and common (R) 5'-GAATTCTTCTGTCAGATGGAGTTACA-3' along with *Kynu* deletion probe FAM-5'-AGCATAGTAACTGCGTGAGATCG-3'-Quencher and *Kynu* wild type probe TAMARA-5'-AGTGGGCCAAGATGTAAGTACC-3'-Quencher. Taqman PCR was performed using Vii7 real-time PCR using Jackson Laboratory’s recommended protocol for genotyping *Kynu* deletion. Animal experiments were conducted with an equal number of age and gender matched (6–10 weeks old male and female) mice. Orthotopic tumors were generated in the indicated mouse line in 6–7-week-old mice. Mice were anesthetized and immobilized using a stereotactic frame, a midline scalp incision was made, and tumor cells (TRP: 1×10^5^ cells/mouse; GL261: 1 × 10^4^ cells/mouse) suspended in 2 µl PBS were injected using a Hamilton syringe localized to specific coordinates (2 mm posterior to bregma, 2 mm mediolateral from the midline, and at a depth of 3 mm). Mice were imaged 6–8 days after implant using Multihance (Bracco Diagnostics, Cranbury, NJ, USA) diluted in sterile saline delivered via tail vein injection under anesthesia. MR imaging were performed as previously described^68^. Briefly, images were acquired from anesthetized mice using a 3.0 T benchtop MRI (MR Solutions, Guildford, UK). Fast spin-echo T2 and T1 weighted images were acquired pre- and post-contrast injection. Tumor volumes were determined by delineating areas of contrast enhancement on the T1 weighted fast spin-echo sequence using the image processing PBAS tool in PMOD software (PMOD Technologies, Zurich, Switzerland). Mice were re-imaged after 2–3 weeks and followed until endpoint criteria were met. GDC-0919 treated mice were performed using oral gavage twice a day (200 mg/kg) for 2 weeks (6 days a week) starting on day 8 following tumor implant. Tumors were isolated on day 25 to analyze QA and KYN levels. Depletion of CD8 T-cells was performed by intraperitoneal injection of 150 μg anti-CD8 (clone: 2.43) mAb on days −3, 10, 17, and 21 after tumor implant. CD8 depletion was verified in parallel in vitro and in vivo experiments.

### Immunophenotyping of tumor resident immune subsets

For analyzing tumor resident immune subsets, we collected orthotopic tumor area from tumor bearing mice. Tumors tissues were dissociated using DNase I and Collagenase IV (Sigma Aldrich, St. Louis, MO) and passed through a 70μM cell strainer to get single cell suspension. Tumor cell suspension was stained with fluorochrome-conjugated mAbs to analyze different subsets using different panels of staining. Using CD45 and CD11b markers alone, all immune cells were clustered into three population (i) macrophages (CD45 + CD11b + ), (ii) lymphocytes (CD45 + CD11b-ve), (iii) microglia (CD45^low^CD11b + )^64^. We further characterized macrophages into M1-like macrophages (CD45 + CD11b + F4/80 + CD206-veCD80^high^), and M2-like macrophages (CD45 + CD11b + F4/80 + CD206 + CD80^low^). In a lymphocyte panel, we analyzed CD4 T lymphocytes (CD45 + CD4 + ), CD8 T lymphocytes (CD45 + CD8 + ), Tregs (CD45 + CD4 + CD25 + FoxP3 + ), and activated CD8 + T lymphocytes (CD45 + CD8 + CD69 + ).

### Immunohistochemical staining

Human TMA sections were obtained from US Biomax (Derwood, MD) and comprised of human astrocytoma grades II, III along with 30 GBM tumors and nine normal brain sections. For mice GBM immunohistochemistry staining, we sacrificed orthotopic GBM mice when the mouse in the experiment presented neurological symptoms of tumor growth. Brains were collected in zinc formalin fixative. The brain was dissected sagittally centered at the syringe hole. The blocks were then processed through graded alcohols and embedded in paraffin. All paraffin-fixed blocks were sectioned from the center at a thickness of four microns. Immunohistochemical staining was performed on FFPE sections deparaffinized and rehydrated using a Leica autostainer, followed by Tris base (pH 9.0) dependent protein antigen retrieval (Vector Labs, Burlingame, CA) at 95 °C. Slides were washed with PBS, incubated in H_2_O_2_ (3% in PBS) for 10 min, washed, and blocked with 5% goat serum in PBS-T (PBS with 0.3% Triton X-100) for 30 min. QA antibody (clone 4E11-G3, 1:500 ImmuSmol, France) was diluted 1:500 in antibody diluent solution (Thermo, Grand Island, NY). Sections were incubated with QA antibody at 4 °C overnight, washed with PBS, and incubated with an HRP-conjugated secondary antibody at room temperature for 1 h. Chromogen DAB (Vector Labs, Burlingame, CA) was applied for 8-10 min after washes. Slides were counter stained using a Leica Autostainer. A section was included in which only the HRP-conjugated secondary antibody was used to serve as a negative control. Sections were imaged using a Aperio Imagescope (Leica Biosystems, Buffalo Grove, IL) at magnification fields (x400). QA staining was quantified by dividing staining intensity into 4 subgroups: strong (3), intermediate (2), low (1), or absent (0). Intensity was multiplied by a factor of 2 if >10 positive foci were observed in each field^69^. Three fields were observed for each TMA sample.

### Statistical analysis

Comparisons across two different groups were performed on original data using 2 tailed student’s T test. One way ANOVA was performed for more than one group, followed by Tukey’s multiple comparisons test. A log-rank test was used for survival analyses. All statistical analyses were performed using Origin Pro 2021 software (Origin Lab Corporation; Northampton, MA).

### Reporting summary

Further information on research design is available in the Nature Portfolio Reporting Summary linked to this article.

## Supplementary information

Supplementary Information

Reporting Summary

## Data Availability

Source data are provided with this paper, which includes raw microarray data associated with Figs. 2b, 4a, and 4b. Access to gene expression data of patient derived gliomas is provided by ArrayExpress under the ID E-MTAB-7116. Data involving metabolomic profiling of human tumors is publicly available and a full dataset can be found in our previous publication^28^. The remaining data are available within the Article, Supplementary Information or Source Data file. Source data are provided with this paper.

## Source data

Source Data
