# Supplementary material for: Quinolinate promotes macrophage-induced immune tolerance in glioblastoma through the NMDAR/PPARγ signaling axis
Source: Nat Commun. 2023 Mar 16;14:1459. doi: 10.1038/s41467-023-37170-z (PMC10020159; doi:10.1038/s41467-023-37170-z)
Supplement: Supplementary file 1 — Supplementary Information [file 41467_2023_37170_MOESM1_ESM.pdf]

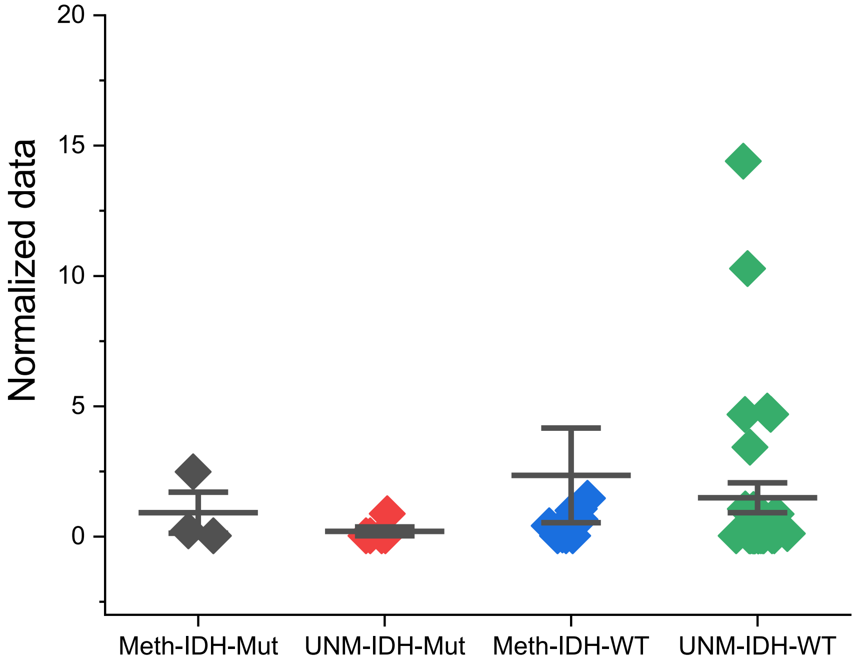

Supplementary Figure 1: QA accumulation in GBM subtypes. The relative accumulation QA as a function of IDH mutation (wild-type [WT] vs. mutant [mut]) and MGMT methylation (methylated [meth] vs. unmethylated [UNM]) status in GBM (Meth-IDH-Mut: n=3; UNM-IDH-Mut: n=5; Meth-IDH-WT: n=14; UNM-IDH-WT: n=34). Y axis abridged at 20. All samples were biologically independent. Data are presented as the mean values ± SEM. Line between data points represents mean, and whiskers represent SE. Line between data points represents mean, and whiskers represent SE. Statistics: One-way ANOVA followed by Tukey's multiple comparisons test (p = not significant). All tests were performed at 95% confidence interval. Source data are provided as a source data file.

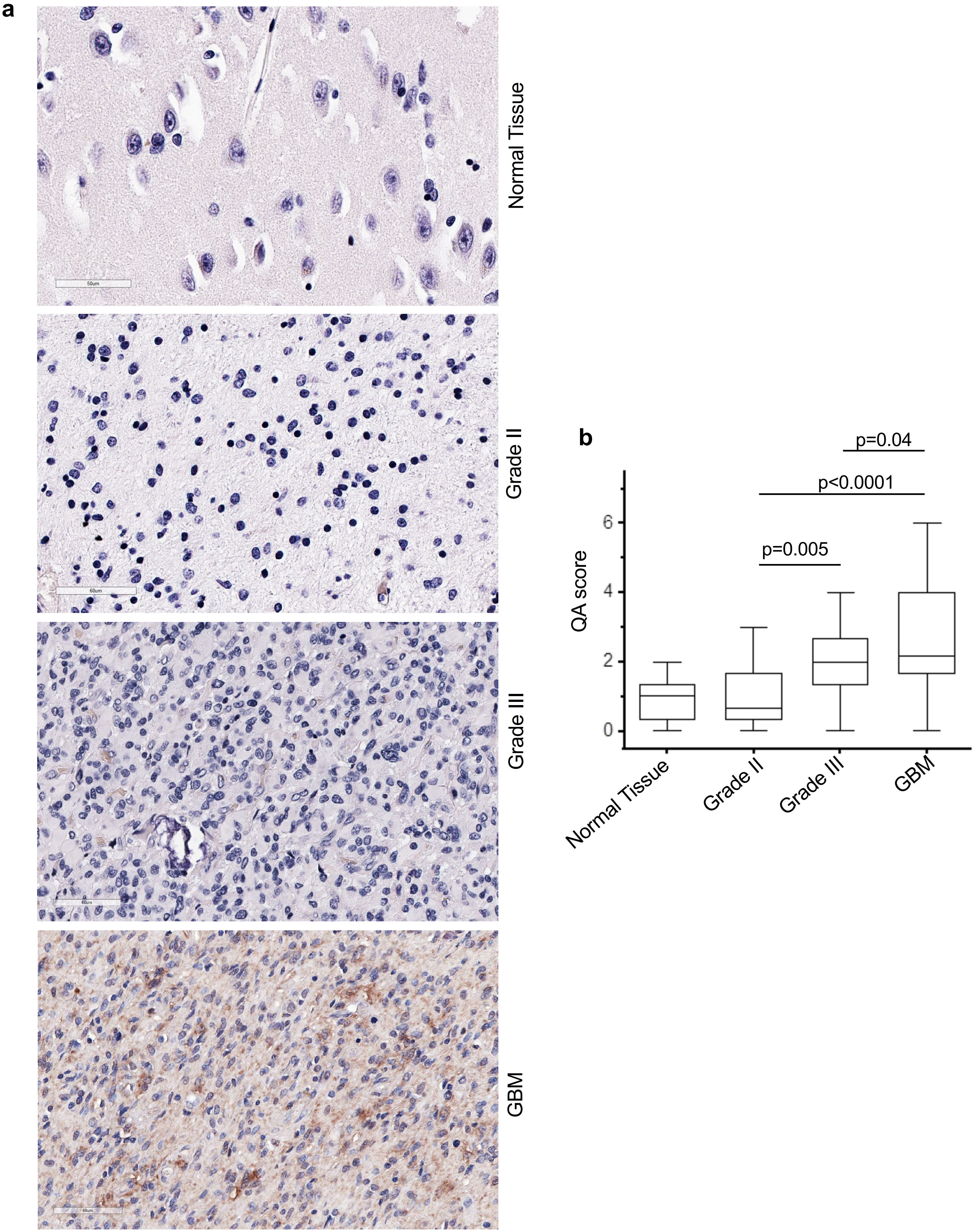

Supplementary Figure 2

Supplementary Figure 2: Immunohistochemical staining of QA in human astrocytoma. (a) Representative depiction of QA immunohistochemical staining (scale bar = 60 μM) and (b) bar graph depicting staining score in normal human brain tissue (n=7), grade II astrocytoma (n=16), grade III astrocytoma (n=12), and GBM (n=31) human tumors. All samples were biologically independent patient samples. In some instances, different regions within the same tumor were included in this tissue array. All available data was used in this analysis. Box plots represent interquartile range, line between data points represents mean, and whiskers represent SE. Statistics: One way ANOVA followed by Tukey's multiple comparisons test (95% CI). Source data are provided as a source data file.

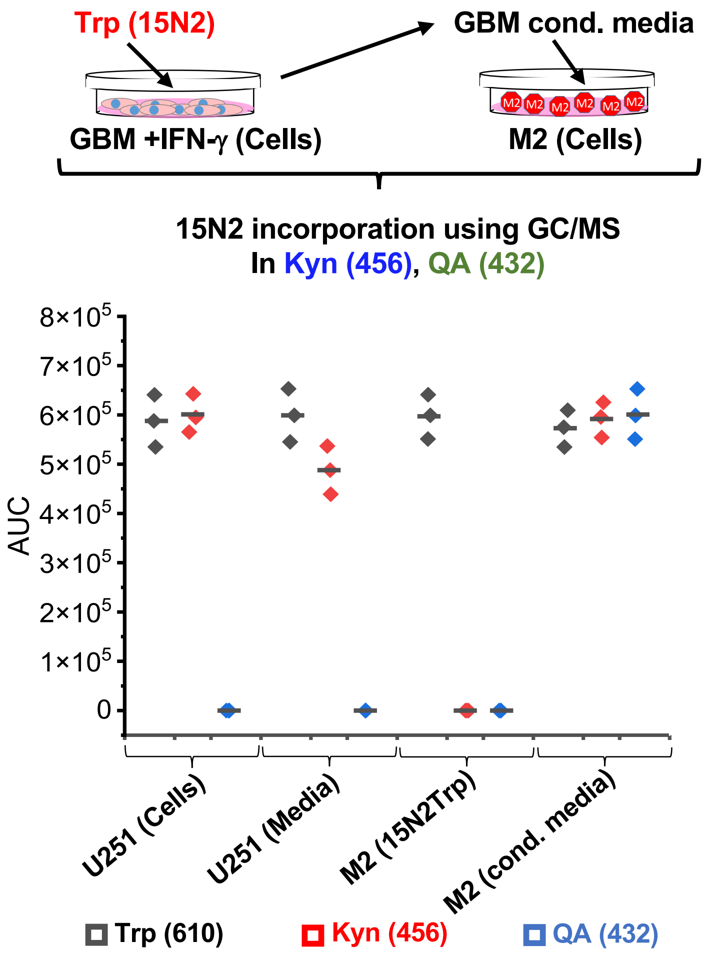

Supplementary Figure 3: Intermediate metabolism of QA in GBM. L-Tryptophan-2,3,3-d3 was used for flux analysis of of Trp→Kyn and Kyn→QA. U251 GBM cells were in culture with labeled tryptophan for 3 days +/- IFNγ. M2 macrophages were then cultured with labeled tryptophan or U251 conditioned media. Trp, Kyn, and QA were then evaluated in U251 cells, U251 media (termed conditioned media), M2 macrophages cultured with labeled tryptophan, or M2 macrophages culture with U251 conditioned media. Labeled QA was only observed in M2 macrophages cultured in U251 media, further supporting the dynamic interactions between tumor and immune cells involved in generating QA. Line between data points represents mean (n=3 biologically independent samples/group). Source data are provided as a source data file.

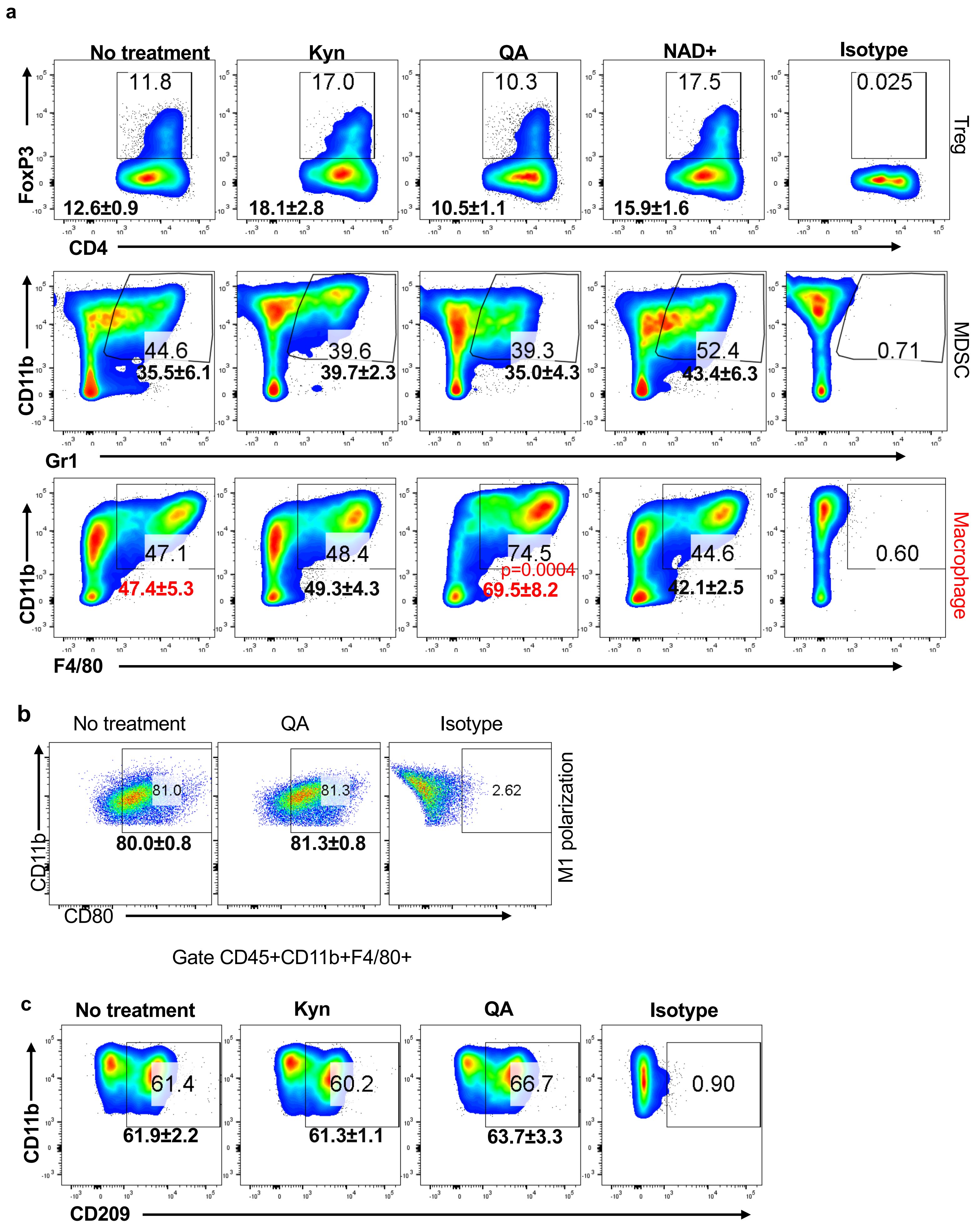

Supplementary Figure 4

Supplementary Figure 4: Influence of QA on immune cells. (a) Tregs (CD4+FoxP3+; n=3), MDSCs (CD45+CD11b+Gr1+; n=4), and (CD45+CD11b+F4/80; n=4) were cultured ± (Kyn- 20μM), QA (20 μM), or NAD+ (20μM) and evaluated by flow cytometry. Tregs were generated by sorting CD4 T from C57BL/6 mouse splenocytes and activating using CD3/CD28 antibodies while MDSCs and macrophages were isolated from mouse bone marrow. All samples were biologically independent. (b) M0 macrophages obtained from the bone marrow of C57BL/6 mice were cultured in GM-CSF (40ng/ml) for 6 days ± QA (20 μM) and polarized to the M1 phenotype with LPS (100 ng/ml) and IFN- γ (50ng/ml) for 24-36 h ± QA (n=3 biologically independent samples/group). Cells were analyzed for M1 macrophage-specific markers (CD45+F4/80+CD11b+CD80hi). (c) Mouse macrophages (n=3 biologically independent samples/group) polarized towards the M2 phenotype (+/- Kyn or QA) were analyzed for presence of CD209+ M2 macrophages (CD45+CD11b+F4/80CD206+CD209+). Numbers represent mean±SD (a, b & c). Statistics: One-way ANOVA followed by Tukey's multiple comparisons test (a, & c), two-tailed Student's *t* test (b). All tests were performed at 95% confidence interval. Source data are provided as a source data file.

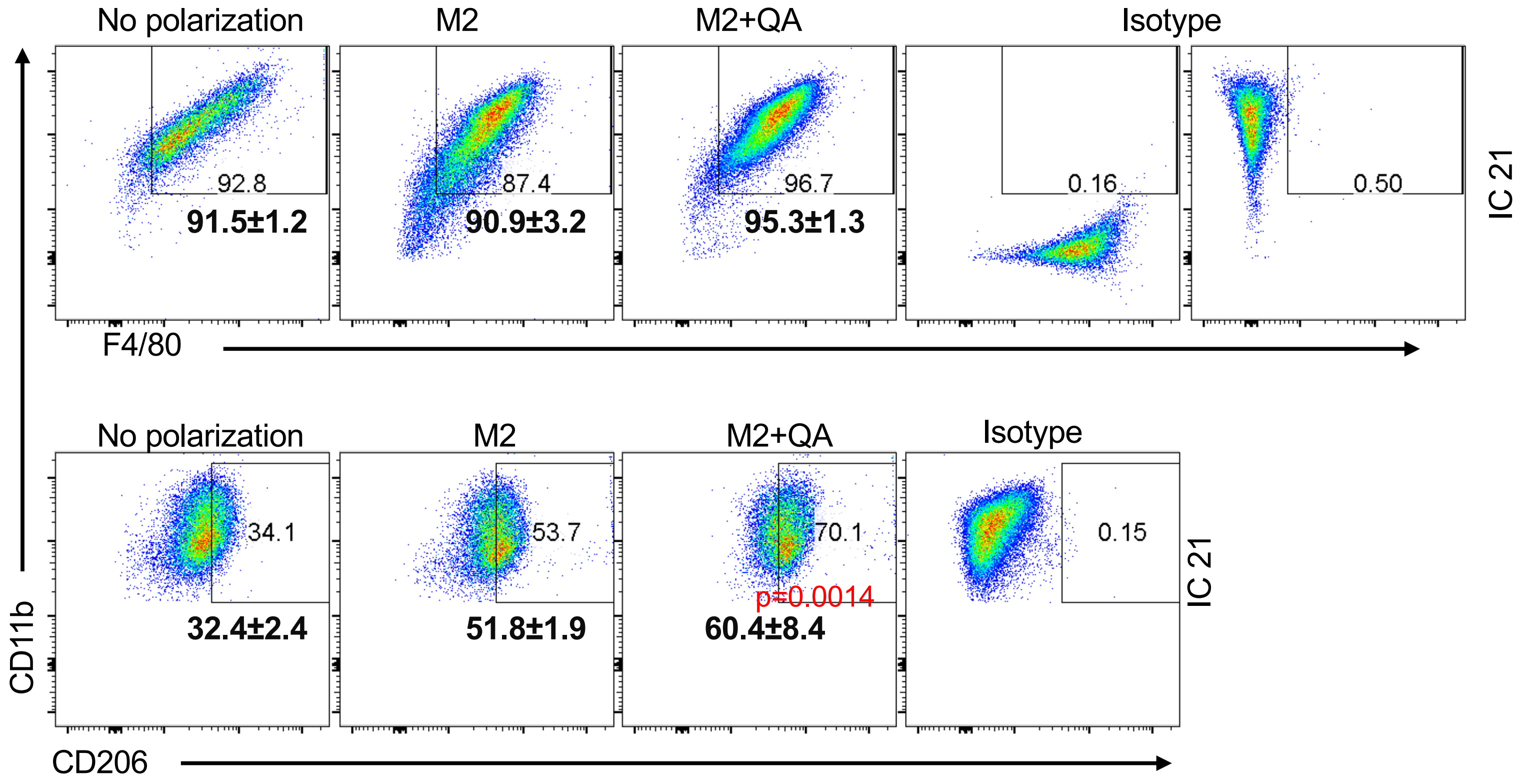

Supplementary Figure 5: The influence of QA on macrophages. The murine macrophage cell line IC-21 was cultured in GM-CSF (40ng/ml) for 6 days ± QA (20 µM). On day 6, cells were re-suspended in IL4 and IL13 (20 ng/ml) for 24 h to polarize towards the M2 phenotype. Cells were analyzed for M2 macrophage markers (CD45+CD11b+F4/80+CD206+). Numbers represent mean±SD (n=3 biologically independent samples/group). Statistics: One-way ANOVA followed by Tukey's multiple comparisons test (95% CI). Source data are provided as a source data file.

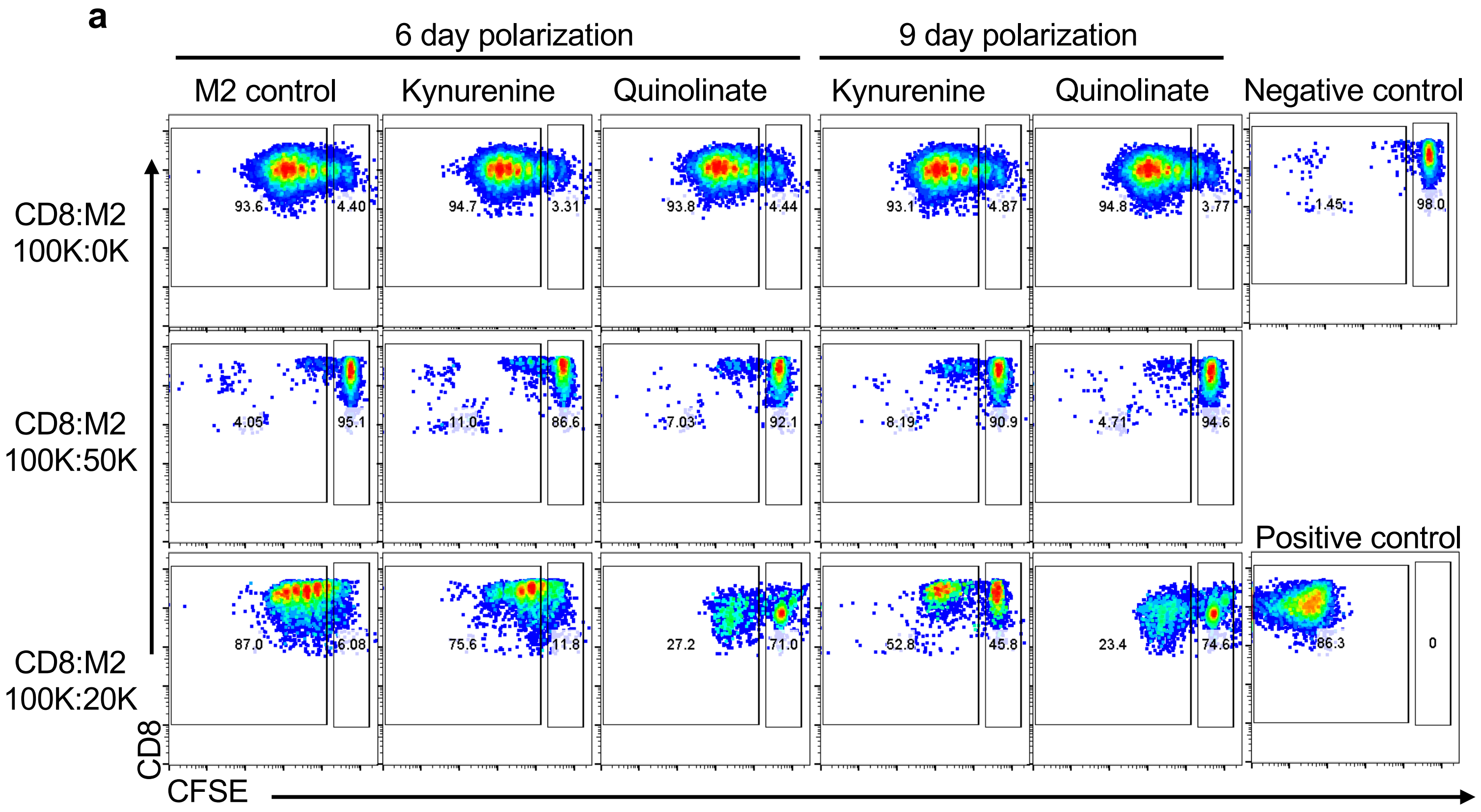

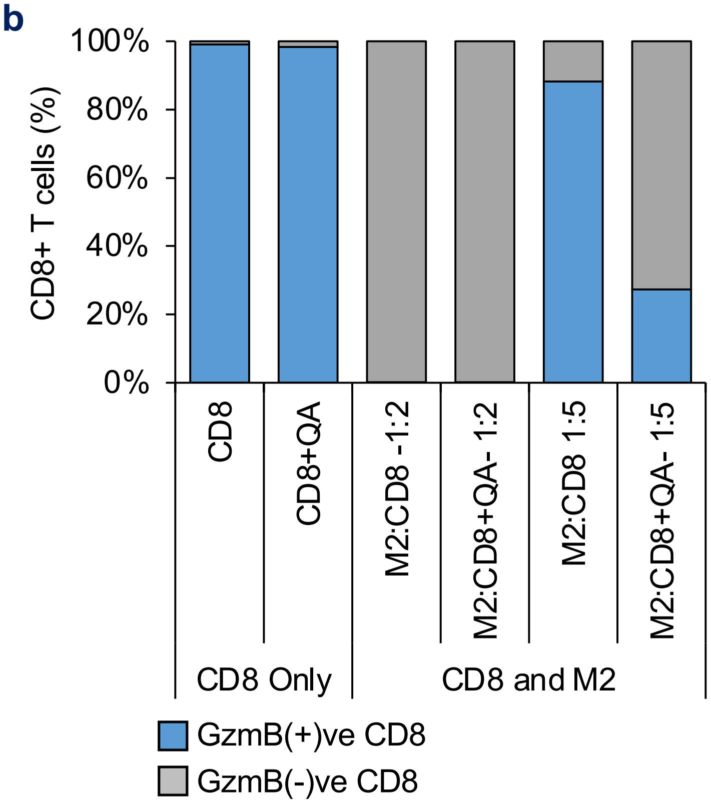

Supplementary Figure 6

Supplementary Figure 6: QA-induced functional suppression of macrophages. This figure is complementary to Fig. 2D, evaluating the suppressive ability of M2 macrophages when evaluated using varying ratios of CD8 T cells. M2 cells were polarized ± Kyn or QA for 6 or 9 d. Splenocytes from C57BL/6 mice were used for isolating $CD8^+$ T cells using magnetic bead sorting. CFSE labeled $CD8^+$ T cells were activated using plate-bound anti-CD3/CD28 antibody for three days in the presence or absence of M2 cells ± Kyn or QA. (a) The proliferation of $CD8^+$ T cells is demonstrated by CFSE dilution. CFSE labeled $CD8^+$ T cells without stimulation (anti-CD3/CD28 antibody) were used as a positive control of suppression. Unlabeled $CD8^+$ T cells were used as a negative control for proliferation. Data is representative of 2 independent experiments. (b) $CD8^+$ T cells were analyzed for granzyme B (GzmB; GzmB+ [gray], GzmB- [blue]). The bar graph represents mean±SD from 2 biologically independent experiments. Source data are provided as a source data file.

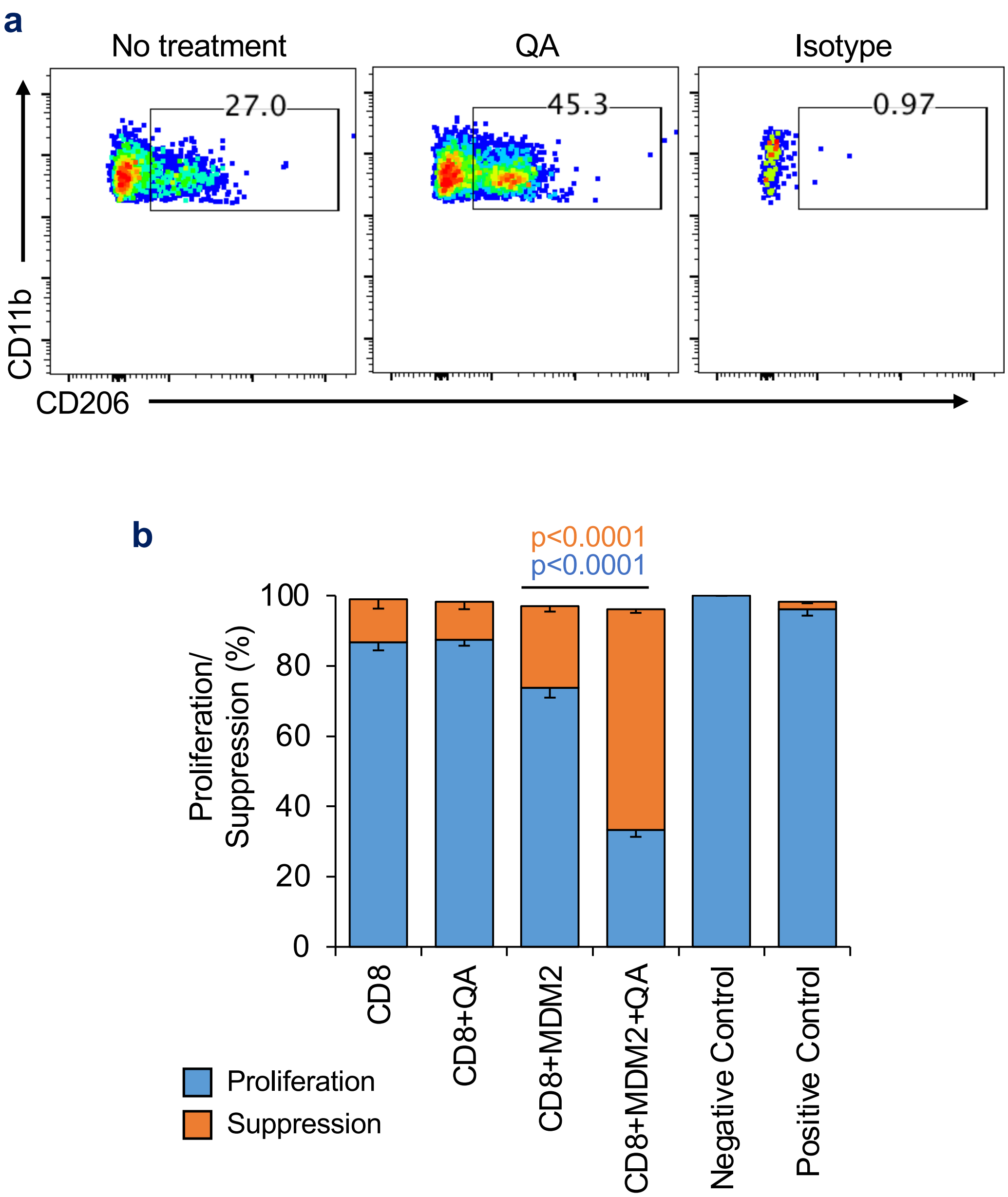

Supplementary Figure 7: QA-induced functional suppression of microglia. (a) Murine microglia derived M2-like macrophages (MDM2) generated from the microglia isolated from brain of C57BL/6 mice were cultured in GM-CSF (40ng/ml) for 7 days in ± QA and polarized to the M2 phenotype with IL4 and IL13 (20 ng/ml) for 48 h to polarize towards the M2-like phenotype. Cells were analyzed for M2 macrophage-specific markers (CD45+F4/80+CD11b+CD206+). Numbers represent mean±SD from 2 biologically independent experiments performed in duplicate. (b) $CD8^+$ T cells (bead sorted from splenocytes of C57BL/6 mice). CFSE labeled $CD8^+$ T cells were activated using plate-bound anti-CD3/CD28 antibody for three days in the presence or absence of MDM2 cells ±QA (n=3 biologically independent samples/group). Proliferation: blue; Suppression: orange. Bar graph shows mean±SD. Statistics: Two-tailed Student's *t* test. All tests were performed at 95% confidence interval. Source data are provided as a source data file.

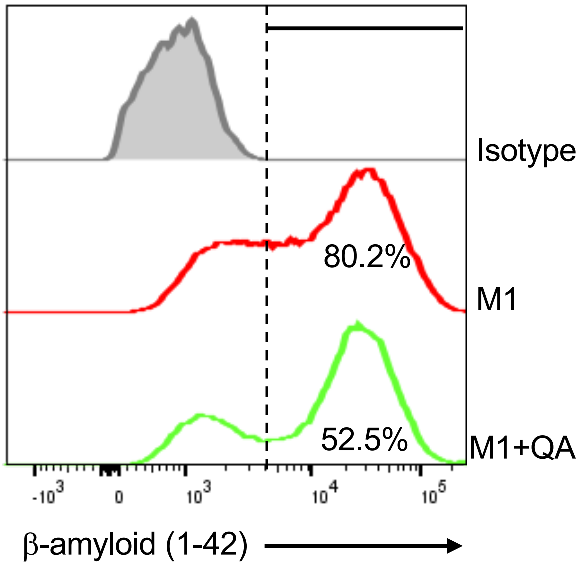

Supplementary Figure 8: QA abrogates phagocytosis in macrophages. Murine M0 macrophages were cultured in the presence of GM-CSF and polarized towards the M1 phenotype (LPS+IFNγ) without (red) or with (green) QA. Cells were pulsed with green florescent β-amyloid (1-42) peptide and analyzed for phagocytosis of this peptide at 16 h. Macrophages were analyzed for the presence of green florescent β-amyloid (1-42) peptide by flow cytometry after gating on M1 specific markers. Results are representative of 2 independent experiments.

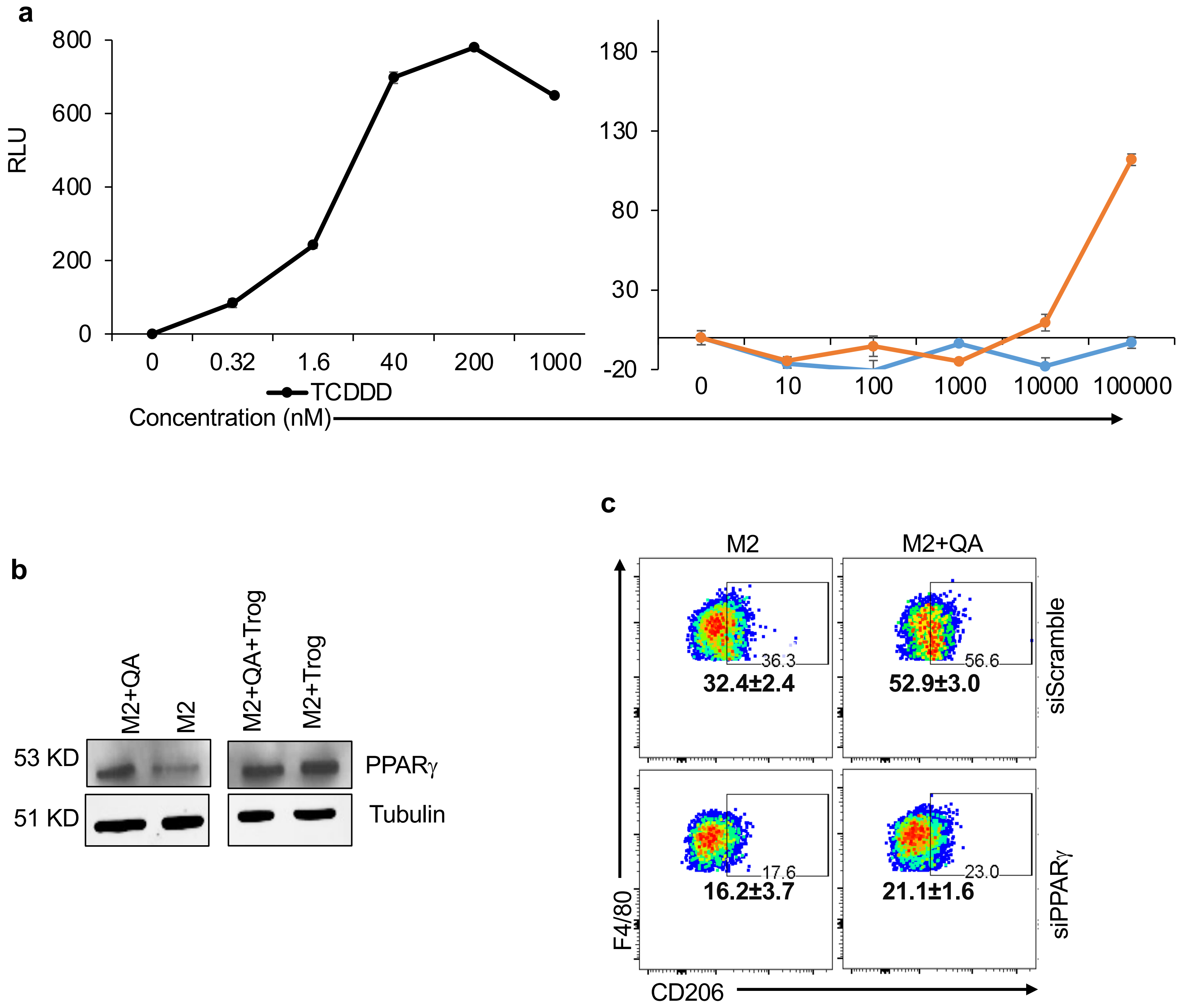

Supplementary Figure 9

Supplementary Figure 9: Mechanisms driving QA-induced changes in macrophage polarization. (a) AhR reporter cells (firefly luciferase, 62 kD protein) were treated with varying concentrations of Kyn (orange), or QA (blue) for 24 hours. 2,3,7,8-tetrachlorodibenzo-p-dioxin (TCDD - ligand of AhR activation) was used a positive control (n=2 experiment/concentration; black). Luciferase was measured using a luminescence reader as relative light units (RLUs). (b) Macrophages obtained from C57BL/6 mice were polarized towards the M2 phenotype ± QA (20 µM) or the PPARγ agonist troglitazone (Trog; 5 µM) and evaluated for the indicated proteins by western blot, which is representative of 3 biologically independent experiments. (c) The murine macrophage cell line IC-21 was cultured in GM-CSF (40 ng/ml) for 5 days ± QA. On day 5 siRNA was used to perform knockdown of PPARγ (siPPARγ). On day 6, cells were re-suspended in IL4 and IL13 (20 ng/ml) for 24 h to polarize towards the M2 phenotype. Cells were analyzed for M2 macrophage markers (CD45+CD11b+F4/80+CD206+). Numbers represent mean±SD (n=4 biologically independent samples/group). Source data are provided as a source data file.

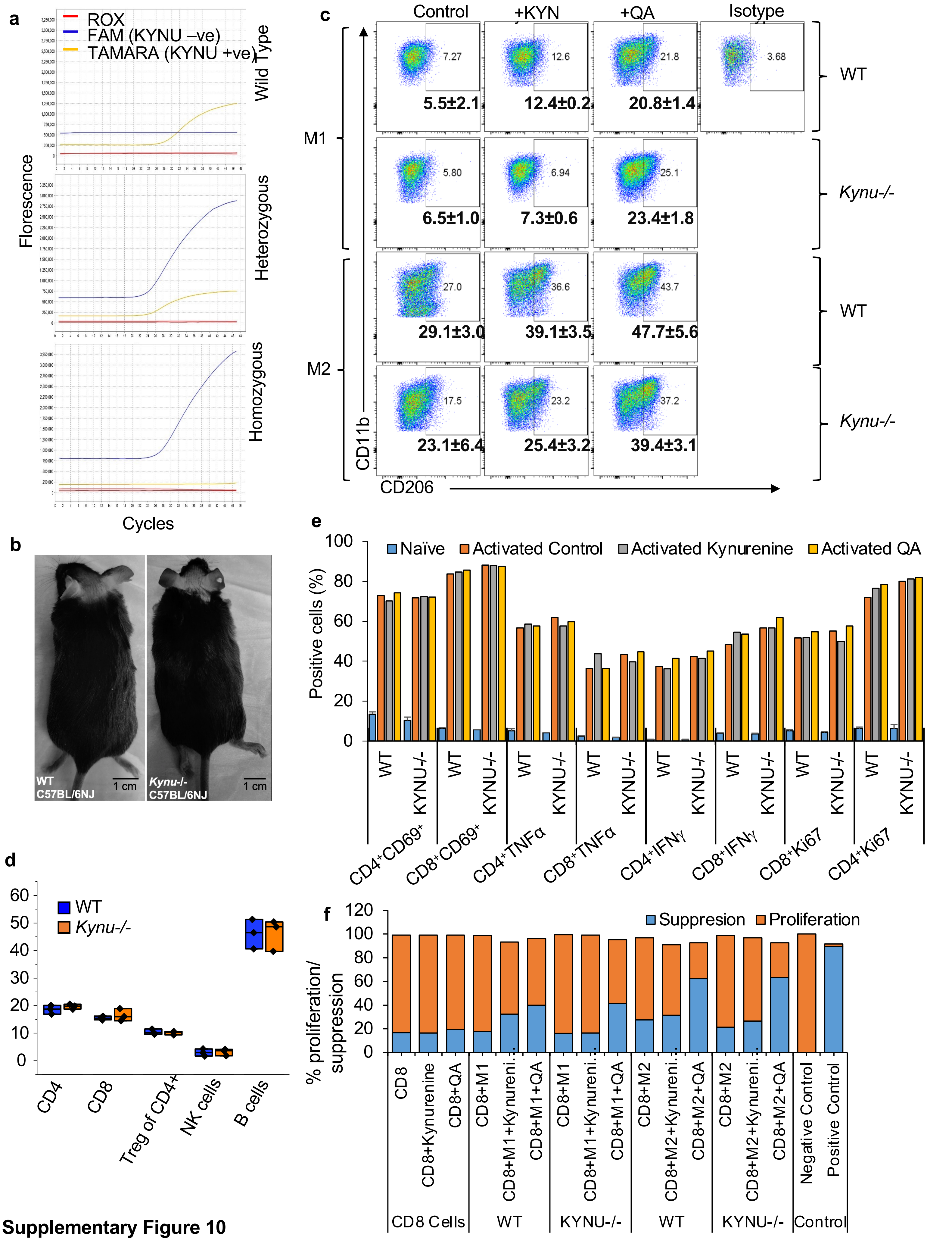

Supplementary Figure 10

Supplementary Figure 10: Validating the *Kynu*-/- mouse. (a) C57BL/6-NJ and *Kynu-/-* mice genotyped using the Taqman PCR assay. FAM probe was used to detect knockout of KYNU, TAMARA Taqman probe detected the wild type KYNU strand. ROX was used as a background control. Each mouse was genotyped in a single experiment and data is representative of the indicated genotype. (b) C57BL/6NJ *Kynu*-/- mice were generated, and phenotype compared to C57BL/6NJ wild type (WT) mice. (c) C57BL/6-NJ and *Kynu-/-* mice were isolated and polarized to M1 or M2 phenotype in ± QA or ± Kyn for 9 days. Cells were analyzed using flow cytometry for the M2 macrophage marker (CD45+CD11b+F4/80+CD206+). Numbers represent mean±SD (n=3 biologically independent samples/group). (d) Naïve splenocytes were isolated from C57BL/6-NJ (WT mice; blue) and *Kynu-/-* mice (orange) and analyzed for $CD4^+$ and $CD8^+$ T cells, T regulatory cells ($CD4^+FoxP3^+CD25^+$), NK cells (NK1.1+), and B cells (CD19+). The bar graph represents mean±SD (n=3 biologically independent samples/group). (e) CD4+ T cells and CD8+ T cells were magnetic bead sorted from C57BL/6-NJ (WT mice) and *Kynu-/-* mice splenocytes and were activated using a plate-bound anti-CD3/CD28 antibody for three days. $CD4^+$ and $CD8^+$ T cells were analyzed for activation marker (CD69) and proliferation marker (Ki67). Activated $CD4^+$ and $CD8^+$ T cells were incubated with PMA/Ionomycin+Protein Transport Inhibitor (Brefeldin A) for 16 hours and analyzed for TNFα and IFNγ (n=2 biologically independent samples/group). (f) Macrophages obtained from C57BL/6-NJ and *Kynu-/-* mice were polarized ± Kyn or ±QA (20 μM) for 9 days. Splenocytes from C57BL/6-NJ mice were used for isolating $CD8^+$ T cells using magnetic bead sorting. CFSE labeled $CD8^+$ T cells were activated using plate-bound anti-CD3/CD28 antibody for three days in the presence or absence of macrophages ± Kyn or QA. The proliferation of $CD8^+$ T cells is demonstrated by CFSE dilution. CFSE labeled $CD8^+$ T cells without stimulation (anti-CD3/CD28 antibody) were used as a positive control of suppression. Unlabeled $CD8^+$ T cells were used as a negative control for proliferation. Proliferation: orange; Suppression: blue. Data is representative of 2 biologically independent experiments. Source data are provided as a source data file.

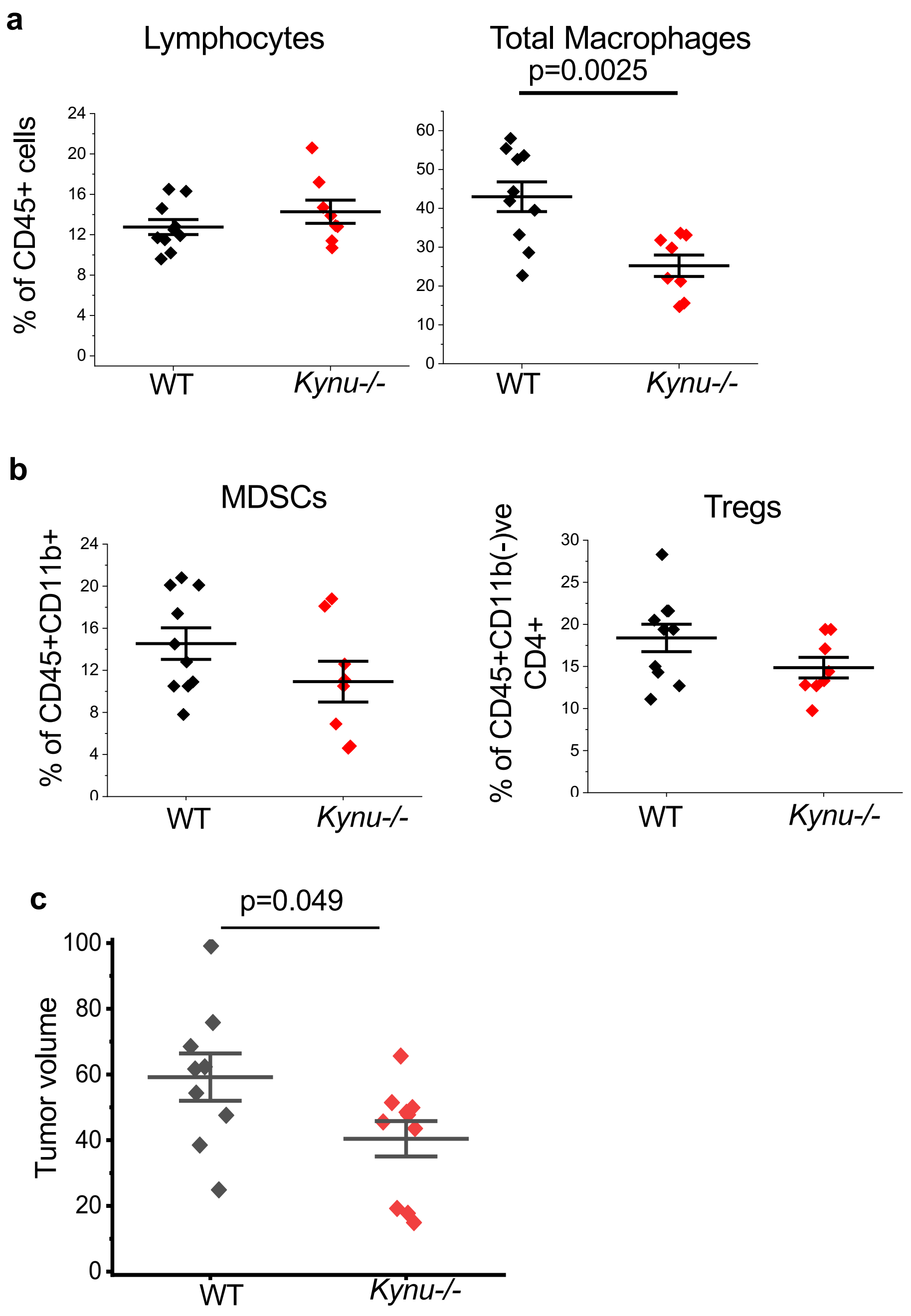

Supplementary Figure 11: Influence of QA on immune cells and tumor growth. TRP tumors were grown orthotopically in C57BL/6-NJ (WT) mice (n=10; black) or *Kynu-/-* mice (n=8; red). (a/b) Tumors were extracted and immunophenotyped for lymphocytes (CD45+CD11b-ve), macrophages (CD45+CD11b+F4/80+), MDSCs (CD45+CD11b+Gr1+) and Tregs (CD45+CD4+FoxP3+CD25+). (c) Tumor volumes were measured using MR imaging on day 21 (n=10/group). All samples were biologically independent. Statistics: Two-tailed Student's *t* test (a, b, & c). All tests were performed at 95% confidence interval. Source data are provided as a source data file.

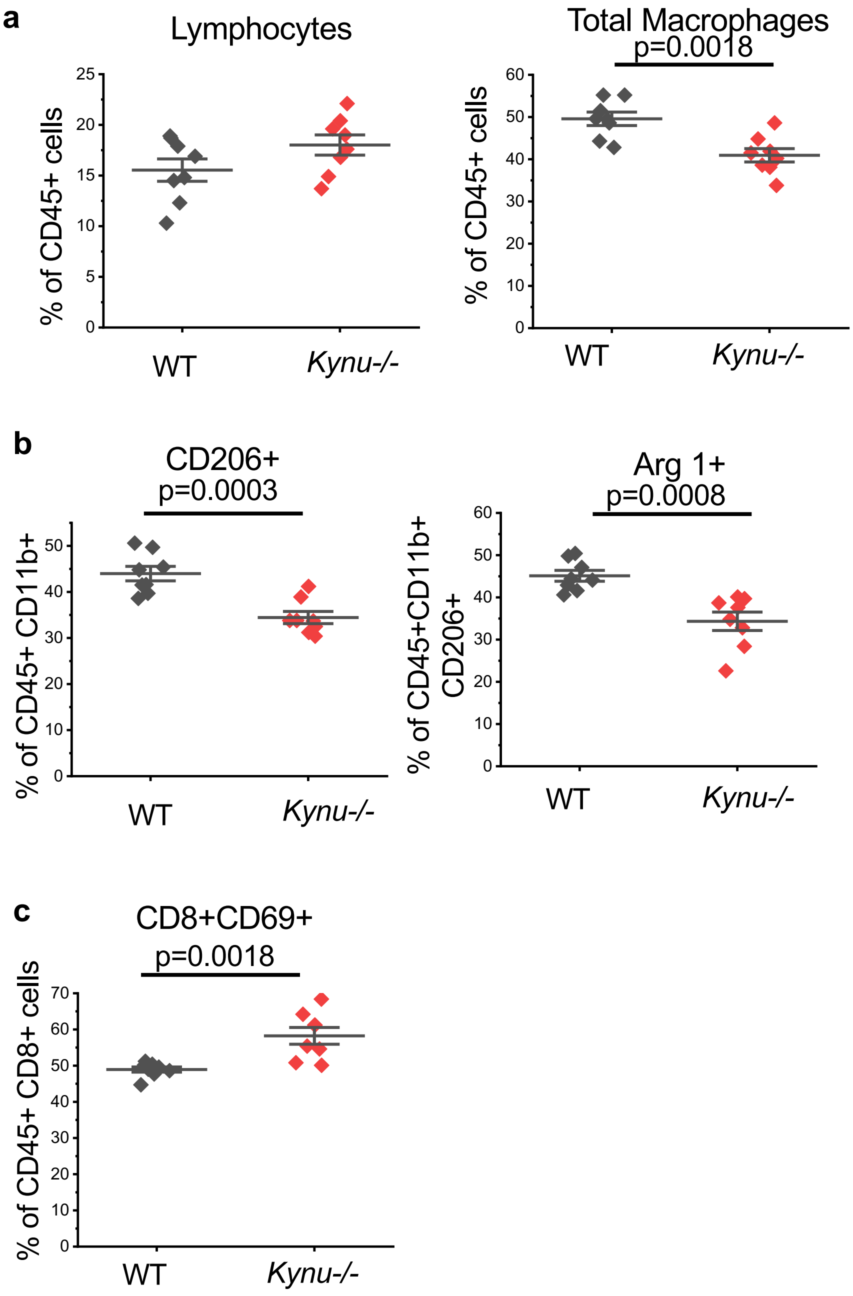

Supplementary Figure 12: GL261 murine GBM tumors were grown orthotopically in C57BL/6NJ WT (black) or *Kynu*-/- mice (red). Mice were euthanized on d 21 and tumors harvested for immune profiling using flow cytometry (n=8 biologically independent samples/group), including (a) lymphocytes (CD45+CD11b-ve); total macrophages (CD45+CD11b+F4/80+); (b) M2 macrophages (CD45+CD11b+F4/801+CD206+); Arginase 1+ M2 macrophages (CD45+CD11b+F4/801+CD206+Arg1+), and (c) activated CD8+ T cells (CD45+CD8+CD69+). Line between the data points represents mean and whisker represents SE. Statistics: Two-tailed Student's *t* test.. All tests were performed at 95% confidence interval. Source data are provided as a source data file.

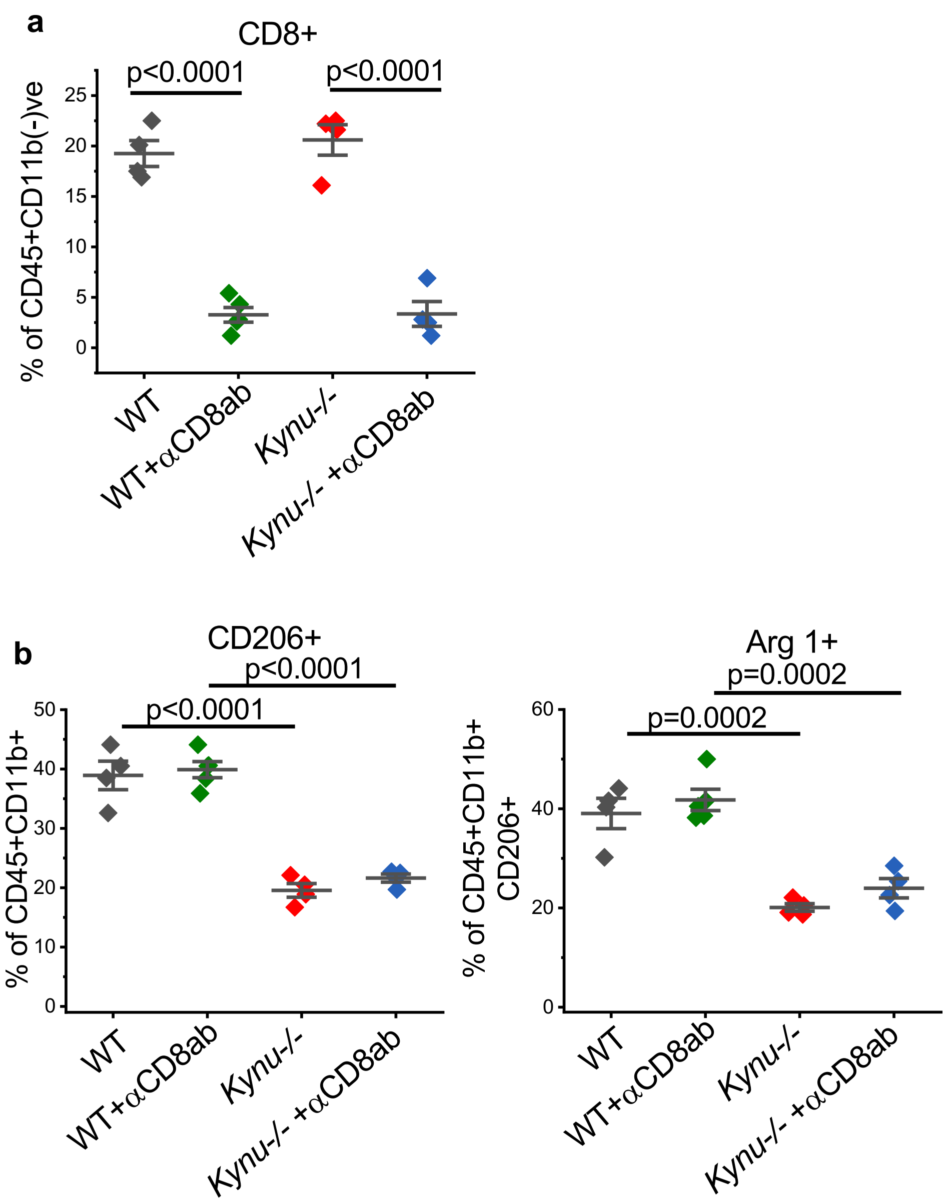

Supplementary Figure 13: Evaluating CD8 cell depletion in *Kynu-/-* mice. TRP tumors were grown orthotopically in C57BL/6-NJ (WT) mice or *Kynu-/-* mice. CD8 T cells depletion was performed in one arm of each group, using anti-CD8 T cell mAb. Tumors were harvested (n=4 or 5 biologically independent samples/group, as indicated in Source Data) on day 21 and used for analyzing (a) CD8+ T cells; (b) M2 macrophages (CD45+CD11b+F4/801+CD206+); and Arginase 1+ M2 macrophages (CD45+CD11b+F4/801+CD206+Arg1+). WT: gray, WT+anti-CD8 T cell mAb (green), *Kynu-/-*: red, Kynu-/- +anti-CD8 T cell mAb (blue). Statistics: One-way ANOVA followed by Tukey's multiple comparisons test (95% CI). Source data are provided as a source data file.

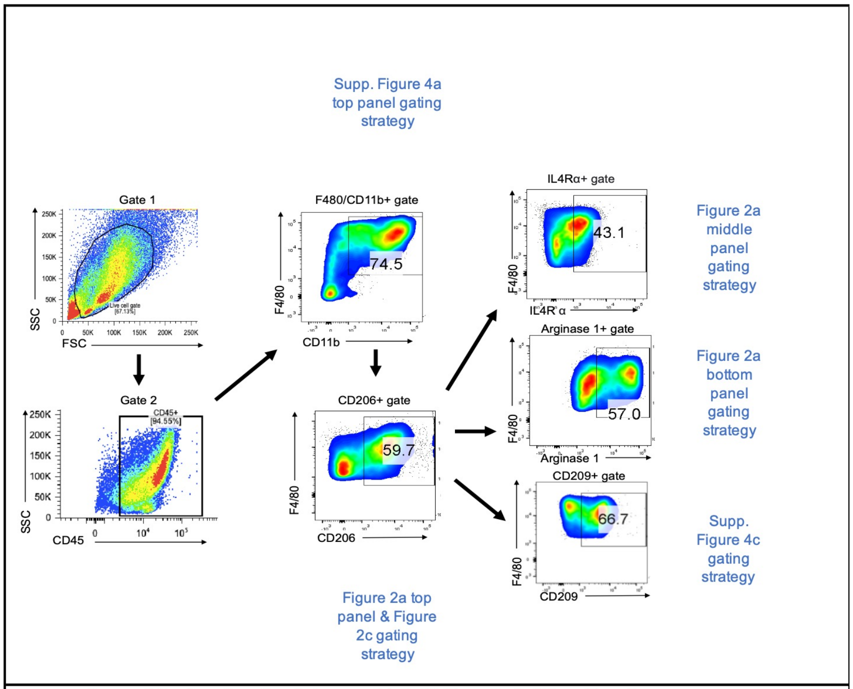

Representative figure for gating strategy associated with Figures 2a, 2c, & Supp. Figure 4a

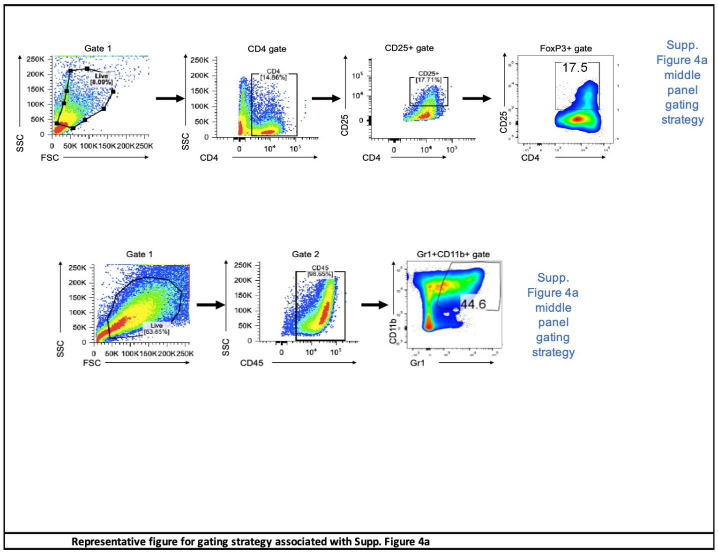

Representative figure for gating strategy associated with Supp. Figure 4a

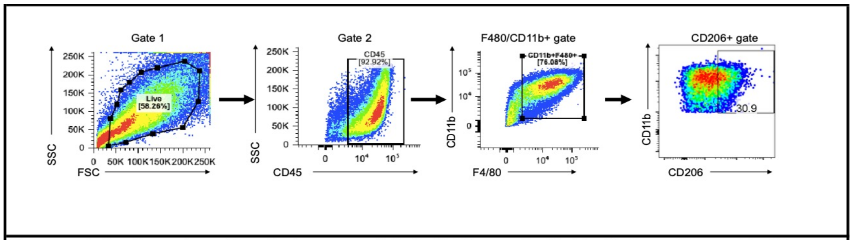

Representative figure for gating strategy associated with Figures 3a, 5b, 5e, 6b, Supp. Figures 7a, 10c

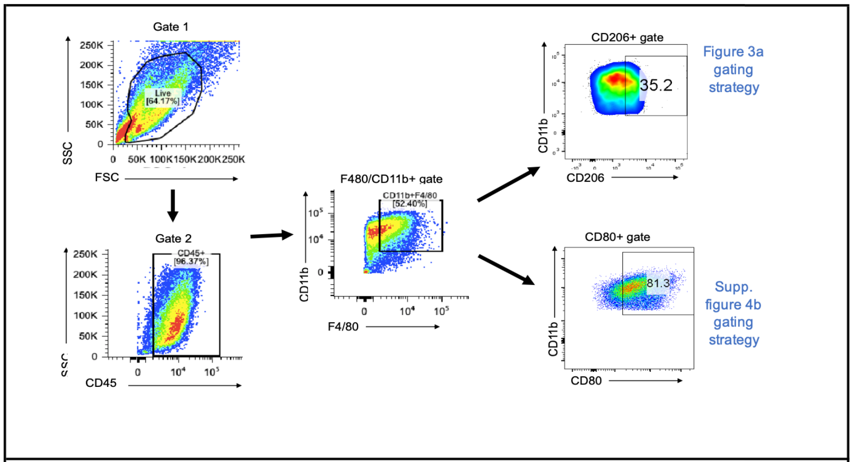

Representative figure for gating strategy associated with Figures 3a, & Supp. Figure 4b

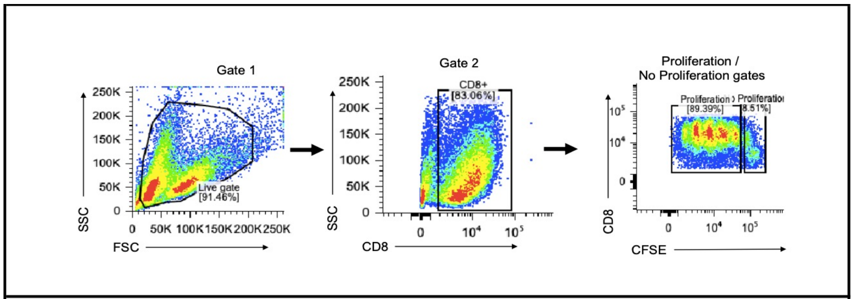

Representative figure for gating strategy associated with Figures 3b, & Supp. Figure 6a

Supplementary Figure 14: Summary of FACS gating/sorting strategies.

Vendor	Antibody	Catalog No.	Clone	Dilution
Biolegend	CD45	103111, 147709	30-F11	1:400
Biolegend	F4/80	123113, 123117	BM8	1:500
eBio/Invitrogen	F4/80	17-4801-80	BM8	1:500
Biolegend	CD11b		M1/70	1:500
Biolegend	CD206	141715, 141705	C068C2	1:200
eBio/Invitrogen	Arg 1	17-3697-82	A1exF5	1:200
Biolegend	IL4Ra		1015F8	1:300
eBio/Invitrogen	iNOS	12-5920-82	CXNFT	1:200
Biolegend	CD209	833003	MMD3	1:400
Biolegend	Gr1	108407, 108423	RB6-8C5	1:400
eBio/Invitrogen	FoxP3	12-4774-41	150/DE4	1:100
Biolegend	CD80	104713	53-6.7	1:500
Biolegend	CD25	102029, 101903	PC61	1:300
Biolegend	CD69	104513	H1.2F3	1:400
Biolegend	GzmB	396409	QA16A02	1:200
Biolegend	GzmB	515403	GB11	1:100
eBio/Invitrogen	Tmem119	25-6119-80	V3RT1G0sz	1:200
Biolegend	CD8a	100711, 100713	53-6.7	1:400
Biolegend	CD4	100406	GK1.5	1:300
eBio/Invitrogen	CD4	25-0041-81	GK1.5	1:300
Biolegend	CD16/CD32	101302	93	1:50
Abcam	NMDAR		N308/48	1:200
Bio X Cell	depletion antinbody CD8	BP0061	2.43	
Eagle Bio/ ImmuSmol	Primary QA antibody	SKU: IS002	4E11-G3	1:500
CST	PPARγ	C26H12		1:1000
CST	Foxo1	C29H4		1:1000
CST	pFoxo1	9461		1:1000
Millipore	Tubulin	2144		1:5000
CST	KYNU			1:2500
Invitrogen	Actin	MA5-11869	C4	1:5000

Supplementary Table 1: Antibody List
